# Comparison of Extracellular Vesicle Isolation Methods for miRNA Sequencing

**DOI:** 10.3390/ijms241512183

**Published:** 2023-07-29

**Authors:** Meritxell Llorens-Revull, Brenda Martínez-González, Josep Quer, Juan Ignacio Esteban, Gonzalo Núñez-Moreno, Pablo Mínguez, Idoia Burgui, Ricardo Ramos-Ruíz, María Eugenia Soria, Angie Rico, Mar Riveiro-Barciela, Silvia Sauleda, María Piron, Irene Corrales, Francesc E. Borràs, Francisco Rodríguez-Frías, Ariadna Rando, Clara Ramírez-Serra, Silvia Camós, Esteban Domingo, Marta Bes, Celia Perales, Maria Isabel Costafreda

**Affiliations:** 1 Liver Diseases-Viral Hepatitis, Liver Unit, Vall d’Hebron Institut de Recerca (VHIR), Vall d’Hebron Barcelona Hospital Campus, Passeig Vall d’Hebron 119-129, 08035 Barcelona, Spain; 2 Centro de Investigación Biomédica en Red de Enfermedades Hepáticas y Digestivas (CIBERehd), Instituto de Salud Carlos III, 28029 Madrid, Spain; 3 Biochemistry and Molecular Biology Department, Universitat Autònoma de Barcelona (UAB), Campus de la UAB, Plaza Cívica, 08193 Bellaterra, Spain; 4 Department of Clinical Microbiology, IIS-Fundación Jiménez Díaz, UAM, Av. Reyes Católicos 2, 28040 Madrid, Spain; 5 Department of Molecular and Cell Biology, Centro Nacional de Biotecnología (CNB-CSIC), Consejo Superior de Investigaciones Científicas (CSIC), Campus de Cantoblanco, 28049 Madrid, Spain; 6 Department of Genetics & Genomics, Instituto de Investigación Sanitaria-Fundación Jiménez Díaz University Hospital, Universidad Autónoma de Madrid (IIS-FJD, UAM), 28040 Madrid, Spain; 7 CIBER de Enfermedades Raras (CIBERER), Instituto de Salud Carlos III, 28029 Madrid, Spain; 8 Bioinformatics Unit, Instituto de Investigación Sanitaria-Fundación Jiménez Díaz University Hospital, Universidad Autónoma de Madrid (IIS-FJD, UAM), 28040 Madrid, Spain; 9 Unidad de Genómica, “Scientific Park of Madrid”, Campus de Cantoblanco, 28049 Madrid, Spain; 10 Centro de Biología Molecular “Severo Ochoa” (CSIC-UAM), Consejo Superior de Investigaciones Científicas (CSIC), 28049 Madrid, Spain; 11 Transfusion Safety Laboratory, Blood and Tissue Bank of Catalonia (BST), 08005 Barcelona, Spain; 12 Transfusional Medicine, Vall d’Hebron Institut de Recerca (VHIR), Universitat Autònoma de Barcelona (UAB), 08035 Barcelona, Spain; 13 Congenital Coagulopathies Laboratory, Banc de Sang i Teixits (BST), 08005 Barcelona, Spain; 14 CIBER de Enfermedades Cardiovasculares (CIBERCV), Instituto de Salud Carlos III, 28029 Madrid, Spain; 15 REMAR-IVECAT Group, Germans Trias i Pujol Research Institute (IGTP), Can Ruti Campus, 08916 Badalona, Spain; 16 Nephrology Unit, Germans Trias i Pujol University Hospital, 08916 Badalona, Spain; 17 Department of Cell Biology, Physiology & Immunology, Universitat Autònoma de Barcelona (UAB), 08193 Barcelona, Spain; 18 Department of Basic Sciences, Universitat Internacional de Catalunya, Sant Cugat del Vallès, 08195 Barcelona, Spain; 19 Clinical Biochemistry Research Group, Vall d’Hebron Research Institute (VHIR), Biochemical Core Facilities, Vall d’Hebron University Hospital, Autonomous University Barcelona, 08035 Barcelona, Spain; 20 Microbiology Department Vall d’Hebron University Hospital, Barcelona Passeig Vall d’Hebron 119-129, 08035 Barcelona, Spain; 21 Clinical Biochemistry Laboratory, ICS-IAS Girona Clinical Laboratory, Doctor Josep Trueta University Hospital, 17007 Girona, Spain; 22 Enteric Virus Laboratory, Department of Genetics, Microbiology and Statistics, School of Biology, Institute of Nutrition and Safety, University of Barcelona (UB), 08007 Barcelona, Spain

**Keywords:** extracellular vesicle isolation, miRNA sequencing, diagnostic biomarker

## Abstract

MicroRNAs (miRNAs) encapsulated in extracellular vesicles (EVs) are potential diagnostic and prognostic biomarkers. However, discrepancies in miRNA patterns and their validation are still frequent due to differences in sample origin, EV isolation, and miRNA sequencing methods. The aim of the present study is to find a reliable EV isolation method for miRNA sequencing, adequate for clinical application. To this aim, two comparative studies were performed in parallel with the same human plasma sample: (i) isolation and characterization of EVs obtained using three procedures: size exclusion chromatography (SEC), iodixanol gradient (GRAD), and its combination (SEC+GRAD) and (ii) evaluation of the yield of miRNA sequences obtained using NextSeq 500 (Illumina) and three miRNA library preparation protocols: NEBNext, NEXTFlex, and SMARTer smRNA-seq. The conclusion of comparison (i) is that recovery of the largest amount of EVs and reproducibility were attained with SEC, but GRAD and SEC+GRAD yielded purer EV preparations. The conclusion of (ii) is that the NEBNext library showed the highest reproducibility in the number of miRNAs recovered and the highest diversity of miRNAs. These results render the combination of GRAD EV isolation and NEBNext library preparation for miRNA retrieval as adequate for clinical applications using plasma samples.

## 1. Introduction

MicroRNAs (miRNAs) are small and highly conserved non-coding RNAs of 20–30 nucleotides in length that play a critical role as post-transcriptional regulators of gene expression [1]. They are involved in biological processes such as development, cell proliferation and differentiation, apoptosis, immune regulation, and metabolism [2]. In addition, they also participate in pathological processes, including primary tumor growth cancer progression, angiogenesis, pre-metastatic niche formation, metastatic cell migration, and anti-cancer drug resistance [3]. MiRNAs are present in many body fluids associated with RNA binding proteins (RBPs), with high and low density lipoproteins, or encapsulated in extracellular vesicles (EVs) [4,5,6]. EVs are lipid membrane vesicles secreted by all cell types [7]. The majority of circulating miRNAs are concentrated in EVs [8]. Furthermore, during a pathological process, injured cells secrete more EVs than healthy cells, and those EVs are enriched with miRNAs involved in the pathogenesis [9].

EVs travel through body fluids, including blood, urine, saliva, bile, ascites fluid, breast milk, semen, and amniotic fluid, and reach distant cells [10]. Three main subtypes of EVs have been described: (i) apoptotic bodies that range in size from 500 nm to 5 µm, (ii) microvesicles (MVs) from 100 nm to 1 µm, and (iii) exosomes from 30 nm to 150 nm, which differ between them in size, origin, content, and function [11]. EVs, and particularly exosomes, serve as functional vehicles of intercellular communication because they carry a complex cargo containing nucleic acids, lipids, and proteins that are capable of modulating and reprogramming recipient cells [12]. Furthermore, the content, size, and membrane composition of EVs vary depending on the cell of origin, its state (e.g., differentiated, stressed, stimulated), and environmental conditions [13]. Relevant differences in the transcriptomic pattern have been observed between EVs and EV-producing cells, indicating a selective incorporation of certain cellular RNAs into the EVs [14], with a selective enrichment of smaller RNAs as compared to the cell transcriptome [15]. Moreover, the RNA cargo of EVs varies between cell types and can also be affected by exogenous stimuli [16,17]. 

The study of miRNA content within circulating EVs rather than total miRNAs in plasma has several advantages. Firstly, EVs provide relevant information on patient status and may offer prognostic information on a broad range of diseases [18]. Secondly, the use of circulating EVs allows real-time information from the different cells involved in the pathological process (e.g., injured cells, immune cells, and metastatic cells) to be compiled [19,20]. Thirdly, the increased stability of the miRNAs enclosed within EVs and their accessibility through biological fluids makes them an attractive alternative as a minimally invasive diagnostic test, known as liquid biopsy [21]. Finally, EVs transfer miRNAs from donor to recipient cells, thereby regulating gene expression locally and distantly during both physiological and pathological processes. Such transfer capability renders EV-associated miRNAs potential diagnostic and prognostic biomarkers as well as potential vehicles for therapeutic agents [22,23]. 

For all these reasons, it is important to have reproducible methods for isolating EVs from biological samples with a high yield. Discrepancies in miRNA patterns are still frequent among studies, which hampers the validation of specific miRNA signatures with diagnostic and/or prognostic value. This is partly due to differences in sample origin and EV–miRNA isolation and sequencing methods [24,25,26]. Moreover, the separation of EVs from other particles such as lipoproteins, which also contain miRNAs, is difficult and varies depending on the isolation method [27]. Hence, EV purification methods that result in minimal contaminants are necessary to enable the precise characterization of miRNA content within EVs. 

Selecting the appropriate isolation method is therefore a critical step for miRNA-based biomarker discovery. We focused our study on miRNAs encapsulated within exosomes since these are the most studied vesicles in cellular communication. However, although most vesicles in the final sample were exosome-like vesicles, herein we will refer to the isolated vesicles as EVs, regardless of the isolation method, considering the difficulty in completely separating exosomes from other EVs [28].

In the present study, we analyzed the yield, abundance, and diversity of miRNAs obtained from the same plasma sample using three different EV isolation methods and three alternative miRNA library preparation protocols for ultra-deep sequencing. The aim of this comparative study was to establish the most suitable methodology to assess circulating miRNA profiles for clinical applications.

## 2. Results

### 2.1. Comparative Characterization of Three EV Isolation Methods

**Figure 1:**
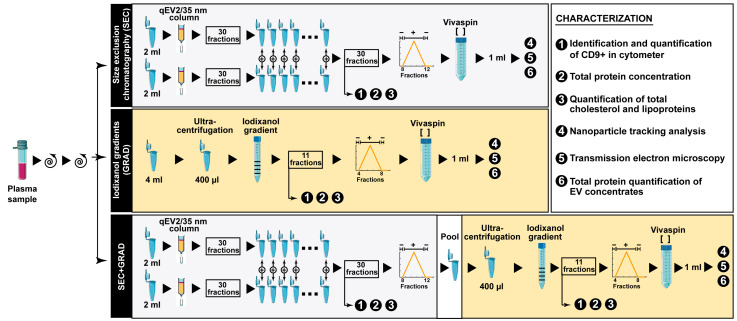
Diagram of EV isolation methods used for the comparison. The box on the right explains the characterization of the products obtained at different steps of the three EV separation protocols. Details are given in Materials and Methods.

To characterize the three EV isolation methods, we measured CD9, apoA, apoB, total cholesterol, and total protein concentration in fractions 1 to 20 for SEC extractions (20 out of the 30 fractions recovered) and all fractions (a total of 11) obtained using GRAD and SEC+GRAD. A schematic of the procedures is shown in Figure 1. Each EV isolation method was assessed in three independent assays.

**Figure 2:**
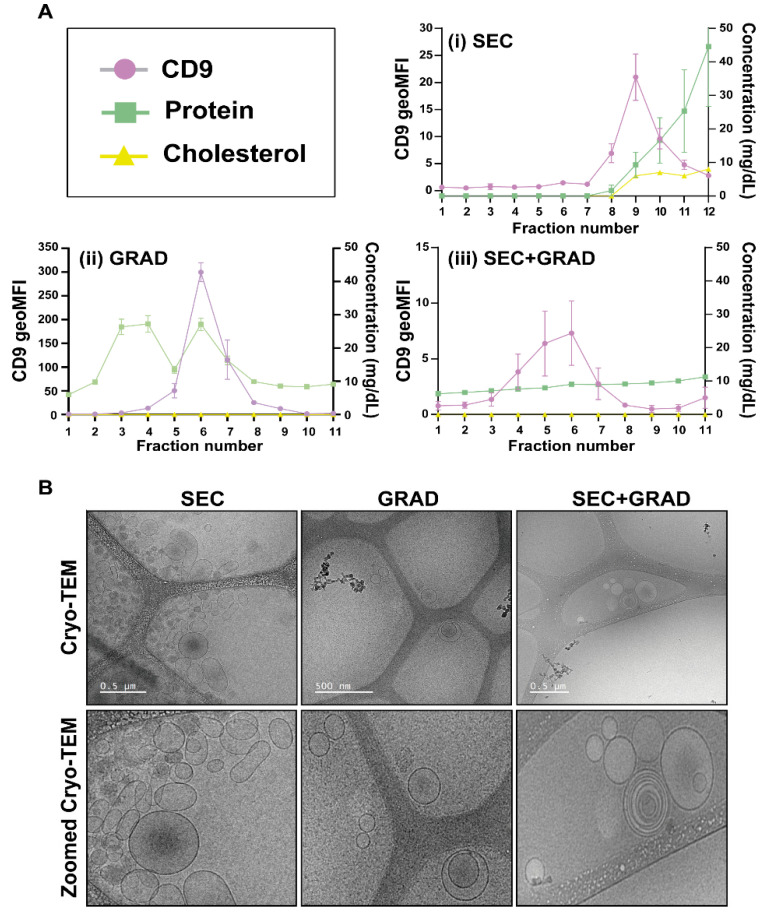
Composition and structure of EVs isolated with three different methods. (**A**) Assessment of CD9, total cholesterol, and total protein in SEC, GRAD, and SEC+GRAD fractions. Codes for the quantifications are depicted in the upper-left box. CD9 geometrical mean fluorescence intensity was measured as geometrical mean was plotted on the left axis. Total cholesterol and total proteins were measured as mg/dL and plotted on the right axis. Total protein was represented as the mean of the three replicas. (**i**) SEC means size exclusion chromatography, (**ii**) GRAD means iodixanol gradients, and (**iii**) SEC+GRAD means size exclusion chromatography followed by iodixanol gradients (see Section 4). (**B**) Cryo-TEM images of EV extractions using SEC, GRAD, and SEC+GRAD. Lower panels (Zoomed Cryo-TEM) are images zoomed in from the corresponding ones shown above.

The tetraspanin CD9 is one of the most common proteins at the EV surface and is therefore considered an EV marker [29]. Because CD9 was consistently detected in SEC, GRAD, and SEC+GRAD fractions that were expected to contain EVs (Figure 2A), CD9 levels were used to monitor the differences between fractionation experiments. ApoA is the major component of high-density lipoprotein (HDL) particles, and it was used as an HDL marker [30]. ApoB is the primary apolipoprotein of chylomicrons, very-low-density lipoprotein (VLDL), intermediate-density lipoprotein (IDL), low-density lipoprotein (LDL), and lipoprotein(a), and it was used as a marker of these lipoproteins [31]. The levels of total cholesterol, apoA, and apoB in the plasma sample were 169 mg/dL, 124 mg/dL, and 102 mg/dL, respectively. In SEC fractions, CD9 tetraspanin reached the highest concentration in fractions 8–12 (Figure 2A), indicating that EVs were mainly located in these fractions, which, in turn, presented low amounts of protein, cholesterol, and lipoproteins (Figure S1). The protein concentration started to rise from SEC fraction 8 and achieved its maximum at fractions 18–19, followed by a decrease in subsequent fractions (Figure S1). An increase in total cholesterol was observed from SEC fraction 9, reaching a plateau between fractions 14 and 16, and decreasing afterwards. A similar trend was followed by HDL, since apoA presented a progressive increase from fraction 16 to 18, with a subsequent decline. We were not able to detect apoB, the marker of chylomicrons, VLDL, IDL, LDL, and lipoprotein(a) in any SEC fraction. In the case of GRAD, fractions 5–8 contained the highest amount of CD9 marker (Figure 2A) and therefore, of EVs. Most proteins were likely pelleted with the GRAD method and the remaining proteins were mainly found in the first fractions of the GRAD and co-purified with those fractions containing the highest CD9 values. Total cholesterol, apoA, and apoB were under the limit of detection (<20 mg/dL, <5.13 mg/dL, and <24.40 mg/dL, respectively). Among the fractions extracted using SEC+GRAD, the highest levels of CD9 fluorescence were detected in fractions 4–7 (Figure 2A). The protein concentration was reduced and showed a slight and continuous increase from the first fraction. Total cholesterol, apoA, and apoB were not detected in any fraction.

The five fractions containing the highest EV concentrations were pooled and concentrated; they correspond to fractions 8–12 for the SEC and 4–8 for the GRAD and SEC+GRAD. The total protein in each EV pool was quantified and compared with the protein content in the original plasma sample, indicating that most of the protein plasma content was removed, with a decrease from about 147 mg/mL to 1.86 mg/mL for the SEC extractions, to 2.02 mg/mL for the GRAD fractionation, and to 1.50 mg/mL for the combination of SEC+GRAD. 

Furthermore, the EV size distribution and concentration of each pool were measured by nanosight (Table 1). The particle size distribution was represented as mean, mode, and percentage of particles present in the sample with the indicated size or smaller (D values; D10, D50, and D90 correspond to the 10%, 50%, and 90% of particles under the reported particle size). Based on the mean concentration of EVs in each pool, we observed that the use of SEC allowed the recovery of a larger amount of EVs than GRAD or SEC+GRAD. Indeed, the mean number of recovered particles/mL with SEC, GRAD, and SEC+GRAD was 7.49 × 10^11^ ± 5.5 × 10^10^, 8.51 × 10^9^ ± 1.98 × 10^8^, and 3.83 × 10^9^ ± 5.53 × 10^8^, respectively. The size of the isolated vesicles was compatible with that of the EVs, especially exosomes, regardless of the method; the size was 133.37 ± 1.93 nm, 136.5 ± 4 nm, and 170.5 ± 4.57 nm with SEC, GRAD, and SEC+GRAD, respectively.

Finally, purified EVs were visualized by cryo-TEM [32] (Figure 2B). In the three images, vesicles with sizes compatible with EVs are observed.

### 2.2. miRNA Library Evaluation for Next-Generation Sequencing

To determine which protocol is more effective for detecting miRNAs in plasma samples, we compared three different protocols for miRNA library preparation: (i) NEBNext Multiplex Small RNA Library Prep Set for Illumina (named as NEB); (ii) NEXTFlex Small RNA-Seq Kit v3 (named as NEXT), and (iii) SMARTer smRNA-Seq Kit (named as SMARTer) (detailed in Section 4). To this aim, we isolated small RNAs from a plasma sample and prepared libraries using the three protocols. We analyzed two replicas with each protocol [Replica 1 (R1) and Replica 2 (R2)]. The resulting RNA libraries were sequenced using Illumina technology (see Section 4).

**Figure 3:**
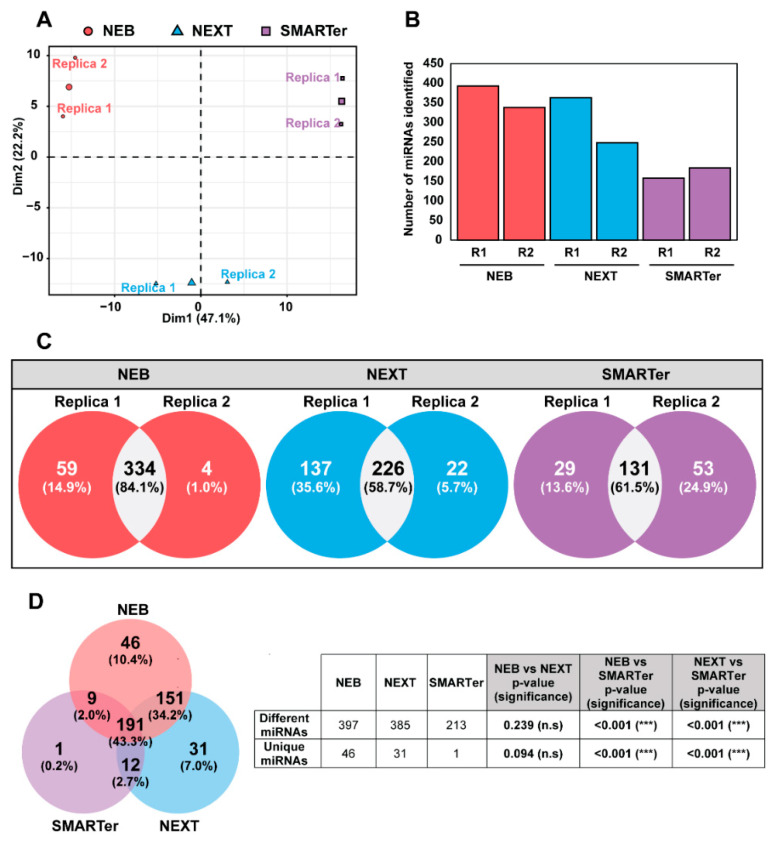
MiRNA profiles vary depending on the miRNA library preparation protocol. NEB refers to NEB Multiplex Small RNA Library Prep Set for Illumina, NEXT refers to NEXTFlex Small RNA-Seq Kit v3 and SMARTer refers to SMARTer smRNA-Seq Kit (see Section 4). (**A**) Principal component analysis (PCA) showing that miRNA profiles are separated based on the library preparation protocol. Each protocol is represented by a different color and symbol. The two replicas are depicted in the plot (Replica 1 and Replica 2). The third point is the centroid of the two replicas of each library protocol. (**B**) Number of different miRNAs identified in each replica of each protocol. (**C**) Venn diagram showing the relationship between the miRNA profiles detected in two replicas of each library preparation protocol. Number and percentage of miRNAs detected in one or two replicas are indicated. (**D**) Venn diagram showing the relationship between miRNA profiles detected with different library preparation protocols. Number and percentage of miRNAs detected by one, two, or three miRNA library preparation methods are indicated. Table showing the number of different and unique miRNAs obtained by the three protocols. A test of proportions comparing the three protocols was performed. *p* values were represented as n.s when no significance was observed and *** when *p* values were <0.001. MiRNA counts with the corresponding accession numbers are listed in Table S2.

Protocols were evaluated considering the following aspects: (i) the recovery of synthetic miRNAs used as controls (called spike-ins), (ii) the reproducibility between replicas, (iii) the number of sequencing reads corresponding to miRNAs, and (iv) the number of different miRNAs detected. Spike-ins were used as controls for the purification and amplification of miRNAs. The number of counts that corresponded with these spike-ins were depicted as a heat map (Figure S2). The three protocols differed between them, with NEXT and NEB being those that showed the highest similarity in spike-in recovery between replicas. Using a principal component analysis (PCA) (Figure 3A), the samples were separated according to the three protocols used when the replicas were plotted together.

Regarding miRNA detection, we selected reads between 17 and 25 nucleotides as miRNAs that were aligned to the human genome and mapped against miRNA coordinates. The total number of reads that corresponds to miRNAs is represented in Table S1. The two replicates sequenced using the NEB and NEXT protocols resulted in a higher number of miRNA reads than the samples processed with SMARTer.

NEB was the protocol that, on average, detected the largest number of different miRNAs, followed by NEXT and SMARTer (Figure 3B and Table S2). Considering the number of different miRNAs, the reproducibility between replicas was analyzed and depicted as Venn diagrams (Figure 3C); NEB was the protocol that showed the highest reproducibility (84.1%) between replicas. The largest number of miRNAs was detected with the NEB protocol (397 out of 441 (90%) different miRNAs found in total, considering the three protocols) (Figure 3D). 

Based on these results, the NEB protocol was selected as the most adequate to prepare a miRNA library from human plasma for subsequent miRNA sequencing.

### 2.3. Selection of an EV Isolation Method According to miRNA Sequence Recovery

**Figure 4:**
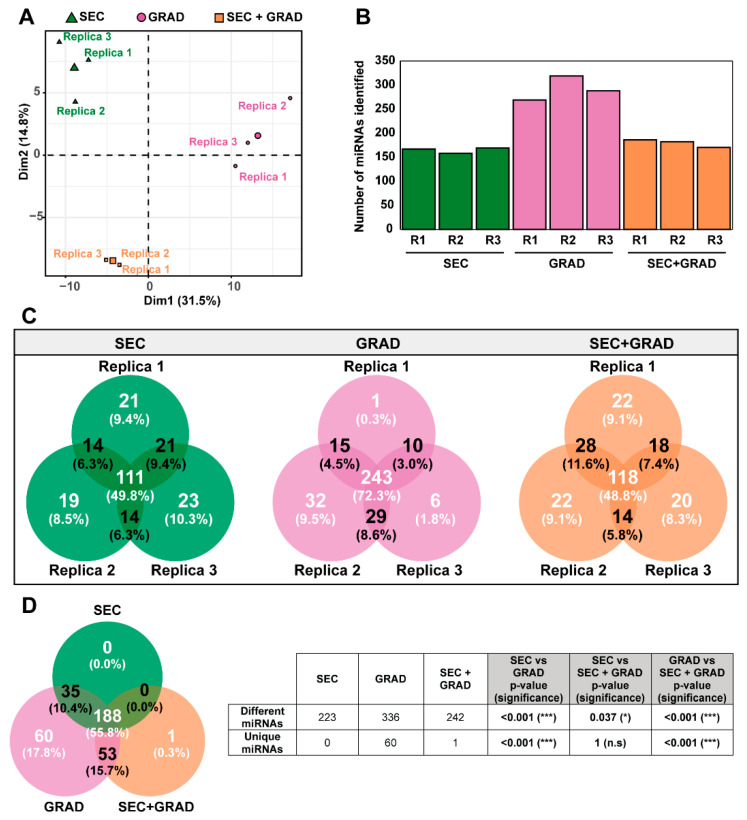
miRNA profiles of circulating EVs vary depending on the EV isolation method. (**A**) Principal component analysis (PCA) showing that miRNA profiles are separated according to the EV isolation method. Each method was assessed in three independent experiments (R1, R2, and R3). The fourth point is the centroid of the three replicas of each EV isolation method. (**B**) Number of different miRNAs detected in replicate experiments (R1, R2, and R3) of each EV isolation method. (**C**) Venn diagram showing the relationship between miRNA profiles of circulating EVs detected in replicate experiments of each EV isolation method. Number and percentage of miRNAs detected in one, two, or three replicas are indicated. (**D**) Venn diagram showing the relationship between miRNA profiles of circulating EVs obtained with different EV isolation methods. Number and percentage of miRNAs detected by one, two, or three EV isolation methods are indicated. Table showing the number of different and unique miRNAs obtained by the three protocols. A test of proportions comparing the three protocols was performed. *p* values were represented as n.s when no significance was observed, * for *p* values ≤ 0.05, and *** for *p* values < 0.001. Counts of miRNAs with the corresponding accession numbers are listed in Table S4. SEC, size exclusion chromatography; GRAD, iodixanol gradient; SEC+GRAD, combination of size exclusion chromatography and iodixanol gradient.

To determine the EV isolation method that allowed detection of more miRNAs, we assessed the miRNA profile of the three independent replicas of each EV isolation method (SEC, GRAD, and SEC+GRAD) using the NEB protocol. Using a principal component analysis (PCA) (Figure 4A), the samples were separated according to the three isolation methods while the replicas were plotted together.

Using the bioinformatics methods previously described [33,34,35], replicas from the GRAD method showed a higher number of miRNA reads than those from SEC and SEC+GRAD (Table S3). GRAD was the method that detected the largest miRNA diversity, followed by SEC and SEC+GRAD (Figure 4B and Table S4).

The reproducibility among replicas was analyzed and depicted as Venn diagrams (Figure 4C); GRAD was the method that showed the highest reproducibility (72.3%) among replicas. The highest number of miRNAs was detected with GRAD (336 out of 337 (99.7%) different miRNAs, considering the three EVs isolation methods) (Figure 4D). The comparison of miRNAs obtained directly from plasma or by the different EV isolation methods showed that GRAD is the procedure that yields the largest percentage of miRNAs shared with the unfractionated plasma sample, followed by SEC+GRAD and SEC (Figure S3).

These results render the GRAD method as one of the most appropriate for detecting miRNAs from EVs isolated from human plasma.

## 3. Discussion

In the present work, we compared different EV isolation and miRNA sequencing methods for the recovery and identification of miRNAs as biomarkers. The choice of an adequate protocol is crucial since it might impact the result of high-throughput differential expression analysis, as documented in previous studies [36]. In addition, easy handling is also a consideration to facilitate routine clinical applicability.

For many years, ultracentrifugation has been used as the gold standard method for EV isolation as it allows the sedimentation of a wide range of vesicles; however, it is a time-consuming procedure that requires expensive equipment and yields low-purity EVs [37]. Precipitation-based methods also result in high EV recovery without specific infrastructure requirements [38], but the low purity of isolated EVs is a major problem. Isopycnic ultracentrifugation using iodixanol gradients has similar requirements as ultracentrifugation in terms of time and equipment, but it provides higher EV purity because it discriminates between vesicle subtypes according to their densities [39]. Finally, size exclusion chromatography is widely used for size-based particle fractionation and yields large EV batches that are well separated from smaller particles and proteins [40].

In this work, we compared three EV isolation methods (SEC, GRAD, and the combination SEC+GRAD). The three procedures resulted in the segregation of vesicles whose sizes are compatible with those of EVs, but they differ in several features, such as the level of lipoprotein and protein contaminants, the EVs recovery yield, the miRNA profiles, the cost-effectiveness, and the clinical applicability. For these reasons, it is important to choose the methodology depending on the intended application of the purified EVs. Regarding the analysis of protein content, the three EV isolation methods removed most of the overabundant soluble plasma proteins, with SEC extractions being those that retained more impurities. Regarding the lipoproteins, we expected no separation between EVs and apoB-containing lipoproteins, such as chylomicrons, LDL, IDL, VLDL and lipoprotein(a), with SEC because of their size overlap (30–5000 nm vs. 18–1200 nm) and no separation between EVs and apoA-containing lipoproteins (HDL) with GRAD due to their density overlap (~1.1 g/mL vs. 1.08–1.19 g/mL) [28]. Nevertheless, all SEC and GRAD EV fractions tested negative for apoA and apoB, suggesting that differential centrifugation along with fractioning methodologies eliminated the vast majority of lipoproteins. However, a certain degree of lipoprotein contamination cannot be excluded based on the physical properties of these particles, which would explain the high quantity of particles detected in the NTA and TEM analyses and the low CD9 fluorescence intensity of the EVs isolated using SEC. As measured by NTA, particle recovery with SEC was two orders of magnitude higher than with GRAD or SEC+GRAD, although the former provided the lowest degree of purity based on TEM and cryo-TEM. Thus, considering CD9 fluorescence intensity as a marker of EV concentration, our results confirmed that GRAD provides a higher proportion of pure EVs than SEC, attaining the best balance between EV purity and yield. 

Additionally, we performed a comparison of the efficacy of three commercially available protocols for library preparation and miRNA sequencing (NEB, NEXT, and SMARTer). In our hands, NEB was the protocol that showed the highest reproducibility between replicas and that resulted in the highest number and variety of miRNAs, followed by NEXT and SMARTer. Our results are consistent with other studies [41,42,43] and reinforce the choice of NEB over other protocols because it is an easy-handling procedure specially applicable to low-input material, as is the case of clinical samples. As a second option, and depending on the source of the input material, the NEXT protocol might be the library preparation of choice [25,44].

The next step was to study the miRNA cargo in the EVs isolated using the three methods. After total RNA level normalization prior to the library preparation and sequencing procedures, GRAD showed the highest reproducibility between replicas and detected the highest number and diversity of miRNAs compared with SEC and SEC+GRAD, indicating that miRNA cargo might vary between EVs isolated with different techniques. We excluded precipitation methods that could also be considered as viable alternatives since they yielded satisfactory recovery of miRNAs from plasma samples in other studies [24]. However, miRNAs related to pathologies that induce small differences in their expression levels and/or are underrepresented in plasma samples can be enriched by isolating the miRNAs within EVs [45]. Our results suggested that the study of miRNAs within EVs isolated by specific methods would allow for the detection of less abundant circulating miRNAs that may play significant roles in pathogenesis and that would be hidden by more abundant miRNAs if alternative methods involving precipitation were used for exosome separation, as was also suggested by other studies [46,47]. The lack of a complete EV characterization is a limitation of our study as it would provide a better understanding of the predominant EV subsets obtained with different techniques. Another study limitation is that the findings of NGS sequencing were not validated by RT-qPCR. However, given the large number of different miRNAs revealed by our methodology (a total of 442), validation based on specific miRNA profiles underlying a clinical outcome seems more appropriate.

In conclusion, all the methods assessed in this study successfully detected miRNAs from plasma samples, although the number of mapped miRNAs varied considerably between EV isolation methods and library preparation kits. In our hands, the GRAD method provided the best balance between EV purity and yield. The NEB library preparation and sequencing kit exhibited the highest reproducibility between replicas; when it was combined with the GRAD EV-isolation method, it resulted in the highest number and diversity of miRNA entities. According to our data, isopycnic centrifugation using the GRAD methodology, followed by NGS sequencing using the NEB kit, should be considered as a reliable method for the identification of miRNA that hold potential as clinical biomarkers.

## 4. Materials and Methods

### 4.1. Plasma Isolation

Plasma was isolated from whole blood from a donor by centrifugation. Plasma aliquots were frozen at −80 °C until use.

### 4.2. Cell Debris, Apoptotic Bodies, and Microvesicle Removal

Plasma samples were centrifuged at 1200× *g* for 20 min at 4 °C. Supernatants were subsequently centrifuged at 10,000× *g* for 30 min at 4 °C, and the clarified supernatants were used for further experimentation. 

### 4.3. Extracellular Vesicle Isolation Methods 

To identify the best EV isolation method intended for miRNA sequencing among those currently available, two different methods and their combination were performed in triplicate and compared (Figure 1). All buffers were filtered with 0.22 μm filters.

Size exclusion chromatography (SEC) was applied to separate vesicles according to their size. qEV2/35 nm columns (Izon Science Ltd., Christchurch, New Zealand) were used for optimal recovery of vesicles between 35 and 350 nm. Two milliliters of clarified supernatants (by two successive centrifugations, as detailed above) were fractionated through the qEV2/35 nm column, following the manufacturer’s instructions. PBS (1X) was used as elution buffer and 30 fractions of 2 mL were collected. The procedure was repeated twice per sample (4 mL each), combining the corresponding fractions. Three independent experiments were performed by using different aliquots of the same plasma sample. 

Isopycnic ultracentrifugation using iodixanol gradients (GRAD) was used to separate vesicles by their densities. Four milliliters of clarified supernatant were concentrated by ultracentrifugation at 100,000× *g* for 3 h at 4 °C using a TH-641 rotor (ThermoFisher Scientific, Waltham, MA, USA). The pellet was re-suspended in 400 µL of PBS, loaded onto an 8–40% iodixanol step gradient prepared with OptiPrep™ (Sigma-Aldrich, Singapore), and centrifuged at 140,000× *g* for 18 h at 4 °C. Eleven fractions of 1 mL were collected from the top of the gradient. The density of each fraction was determined by the absorbance of iodixanol at 240 nm.

The combination of SEC and GRAD (SEC+GRAD) was used to separate vesicles by size and density. EVs from 4 mL of clarified supernatant were purified using SEC as described above. Then, fractions containing EVs were pooled, concentrated, and purified by isopycnic ultracentrifugation as above. 

### 4.4. EV Characterization

For EV marker detection, 50 µL of SEC fractions containing EVs or 12.5 µL of GRAD and SEC+GRAD fractions (fractions 1 to 15 and 1 to 11, respectively) were mixed with 0.5 µL of aldehyde/sulfate-latex beads, 4% *w*/*v*, 4 µm (ThermoFisher Scientific), and PBS up to 55 µL and incubated for 15 min at room temperature (RT). Non-specific binding was blocked with 5% filtered BSA Blocker™ (Thermo Scientific™) overnight at RT in an orbital shaker. CD9 tetraspanin staining was performed with the primary antibody Pure Anti-Human CD9 (Immunostep SL, Salamanca, Spain) at RT for 30 min, washed, and subsequently stained with the FITC-labeled Goat F (ab’)2 Anti-Mouse IgG (H+L) secondary antibody (SouthernBiotech, Birmingham, AL, USA) for 30 min at RT in darkness (the protocol has been adapted from [48]). For the identification and quantitation of CD9+ subsets, 100,000 events were acquired per sample in a FACSCalibur cytometer (BD Biosciences, San Jose, CA, USA). FlowJo software version 10.8.1 (BD Biosciences, San Jose, CA, USA) was used for data analysis. 

Total protein concentration was measured using the Thermo Scientific™ Pierce™ BCA assay kit (Thermo Scientific™). In brief, 25 µL of the first 20 fractions from SEC or 6.25 µL of all gradient fractions in PBS up to 25 µL were incubated with BCA reagent and 4% cupric sulfate at 37 °C for 30 min in darkness. Absorbance was measured at 562 nm on a Varioskan™ LUX multimode microplate reader (Thermo Scientific™).

Total cholesterol was measured using the AU5800 (Beckman Coulter, Singapore), and apolipoprotein A (apoA) and apolipoprotein B (apoB) were determined by nephelometry in the automated system BN II (Siemens healthcare, Erlangen, Germany).

Fractions containing EVs isolated using SEC, GRAD, or SEC+GRAD were pooled and concentrated down to 1 mL with Vivaspin^®^ 20 Ultrafiltration Unit of 100 K MWCO PES (Sartorius, Göttingen, Germany), following the manufacturer’s instructions. Total protein in EV concentrates was quantified by using the BCA method. Size and concentration of purified EVs were assessed with a nanoparticle tracking analysis (NTA) using the Nanosight N300 instrument (Malvern Panalytical, Singapore). Morphological analyses of the EVs in each concentrate were performed by transmission electron microscopy (TEM) and cryo-TEM.

### 4.5. RNA Extraction

RNA was extracted from 250 µL of the plasma sample or from 250 µL of EV isolation samples using a miRNeasy Mini Kit (QIAGEN, Singapore), following the manufacturer’s instructions. Purified RNA was eluted in 20 μL of nuclease-free water. To monitor the purification and amplification of miRNAs, 52 spike-ins (synthetic miRNAs at different concentrations) (QIAseq miRNA Library QC PCR Assay Kit) were added to the initial sample before RNA extraction. Quantity and quality of the purified RNA were determined using NanoDrop and Bioanalyzer 2100 (Agilent Technologies, Santa Clara, CA, USA), respectively; the RNA concentrations ranged between 164 pg/µL and 349 pg/µL.

### 4.6. Library Preparation, Size Selection, and Sequencing

Libraries of small RNA were prepared using three different protocols: (i) NEBNext Multiplex Small RNA Library Prep Set for Illumina (New England BioLabs, Ipswich, MA, USA); (ii) NEXTFlex Small RNA-Seq Kit v3 (PerkinElmer, Waltham, MA, USA); and (iii) SMARTer smRNA-seq kit (Clontech Laboratories, Mountain View, CA, USA), according to the manufacturer’s guidelines. The amount of RNA was 0.7 ng for the plasma 1 sample and 1 ng for the plasma 2 sample. The size profiles of the individual libraries were analyzed using a high sensitivity DNA assay on a Bioanalyzer 2100 (Agilent Technologies, Santa Clara, CA, USA). Size selection of fragments was carried out using 5% Mini-PROTEAN TBE Gel pre-cast polyacrylamide gel (Bio-Rad Laboratories, Hercules, CA, USA). Electrophoresis was performed at 120 V for 1 h in tris-borate-EDTA (TBE) buffer. The band of interest was eluted, following the NEXTFlex kit guidelines. Libraries were quantified by qPCR using KAPA Sybr fast master mix (2X) optimized for LightCycler 480 (KAPA Biosystems, Wilmington, MA, USA) and an in-house standard library from Fundación Parque Científico de Madrid. Quantified libraries were mixed at equimolar ratios and sequenced with a NextSeq 500/550 High Output Kit v2.5 (75 Cycles) using the NextSeq 500 system (Illumina, San Diego, CA, USA).

### 4.7. Bioinformatics Analyses

Read trimming was performed with Cutadapt (version 3.2) [35], following the guidelines of each library preparation kit. Samples prepared with the NEXTflex kit were trimmed by removing the 3′ adapter ′TGGAATTCTCGGGTGCCAAGG′ plus 4 bases from both ends of each read. The TruSeq 3′ adapter ′AGATCGGAAGAGCACACGTCTGAACTCCAGTCA′ was trimmed from samples prepared with the NEBNext kit. Samples prepared with SMARTer were trimmed by removing the 3′ polyA adapter (′AAAAAAAAAA′) plus 3 extra bases at the 5′ end.

After adapter trimming, reads with lengths between 17 and 25 nucleotides were selected and mapped to the hg38 reference genome using default parameters using Bowtie 2 (version 2.3.4.3) [34]. This corresponds to mature miRNAs. Quantification was performed using HTSeq (version 0.13.5) [33] with default parameters using mature miRNA coordinates provided by miRBase v22.1 (https://www.mirbase.org/ftp/22.1/genomes/hsa.gff3, accessed on 18 June 2021). Low expressed miRNAs (those with less than 50 reads in total) were discarded. Principal component analysis was performed using TMM normalized counts.

For spike-in analysis, selected reads between 17 and 25 nucleotides were mapped to the QIAseq™ miRNA Library QC Spike-In sequences using Bowtie 2 (version 2.3.4.3). Mapping parameters were tuned for “perfect match” as recommended in the QIAseq™ miRNA Library QC Spike-Ins protocol by setting the following parameters: “--end-to-end -N 0 --mp 10000 --np 10000 --rdg 10000 --rfg 10000”. Mapped reads were quantified and normalized by dividing them by the total number of mapped spike-ins per sample. Heatmaps were plotted using the “pheatmap” R package over the log2 normalized values.

### 4.8. Statistics

The statistical significances of different comparisons were calculated by the proportion test, using software R version 4.0.2. *, *p* < 0.05.

## Figures and Tables

**Table 1:** NTA parameters of each replica.

EV Isolation Methods	Concentration (Particles/mL)	Mean (nm)	Mode (nm)	D10 (nm)	D50 (nm)	D90 (nm)
*SEC1*	1.29 × 10^12^ ± 1.24 × 10^11^	128.4 ± 1.5	96.4 ± 5.4	80.9 ± 2.1	116.2 ± 1.3	194.1 ± 2.1
*SEC2*	5.63 × 10^11^ ± 3.89 × 10^10^	134.5 ± 2.4	108.4 ± 4.1	87.6 ± 0.8	121.2 ± 1.6	206.7 ± 8.5
*SEC3*	3.93 × 10^11^ ± 2.22 × 10^9^	137.2 ± 1.9	114.8 ± 7.7	78.3 ± 2.3	124.2 ± 1.8	215.7 ± 3.4
*GRAD1*	7.29 × 10^9^ ± 8.16 × 10^7^	134.7 ± 1.2	95.5 ± 1.1	86.1 ± 2.8	120.6 ± 1.2	200.9 ± 4.7
*GRAD2*	9.55 × 10^9^ ± 1.95 × 10^8^	133.7 ± 1.6	94.3 ± 2.5	82.8 ± 1.4	120.2 ± 1.8	203.7 ± 3.3
*GRAD3*	8.68 × 10^9^ ± 3.18 × 10^8^	141.1 ± 9.2	95.1 ± 4.0	85.3 ± 5.0	123.7 ± 6.7	215.9 ± 14.0
*SEC+GRAD1*	4.90 × 10^9^ ± 2.23 × 10^8^	164.2 ± 2.7	129.1 ± 8.1	94.1 ± 2.9	146.2 ± 3.2	259.4 ± 11.2
*SEC+GRAD2*	4.30 × 10^9^ ± 1.31 × 10^9^	179.7 ± 7.3	140.4 ± 28.7	96.1 ± 3.9	161.0 ± 14.4	277.6 ± 12.5
*SEC+GRAD3*	2.29 × 10^9^ ± 1.25 × 10^8^	167.6 ± 3.0	111.5 ± 2.5	94.9 ± 9.0	148.3 ± 2.9	278.9 ± 16.8

## Data Availability

Fastq files of samples included in this study are available in GEO under the BioProject ID PRJNA928492 with the following references: GSM6997624-GSM6997638.

## Supplementary Materials

The supporting information can be downloaded at: https://www.mdpi.com/article/10.3390/ijms241512183/s1.
