# Supplementary material for: Comparison of Extracellular Vesicle Isolation Methods for miRNA Sequencing
Source: Int J Mol Sci. 2023 Jul 29;24(15):12183. doi: 10.3390/ijms241512183 (PMC10418926; doi:10.3390/ijms241512183)
Supplement: Supplementary file 1 [file ijms-24-12183-s001.zip › ijms-2458258-supplementary.pdf]

Supplementary material for:

Comparison of extracellular vesicle isolation methods for miRNAs sequencing

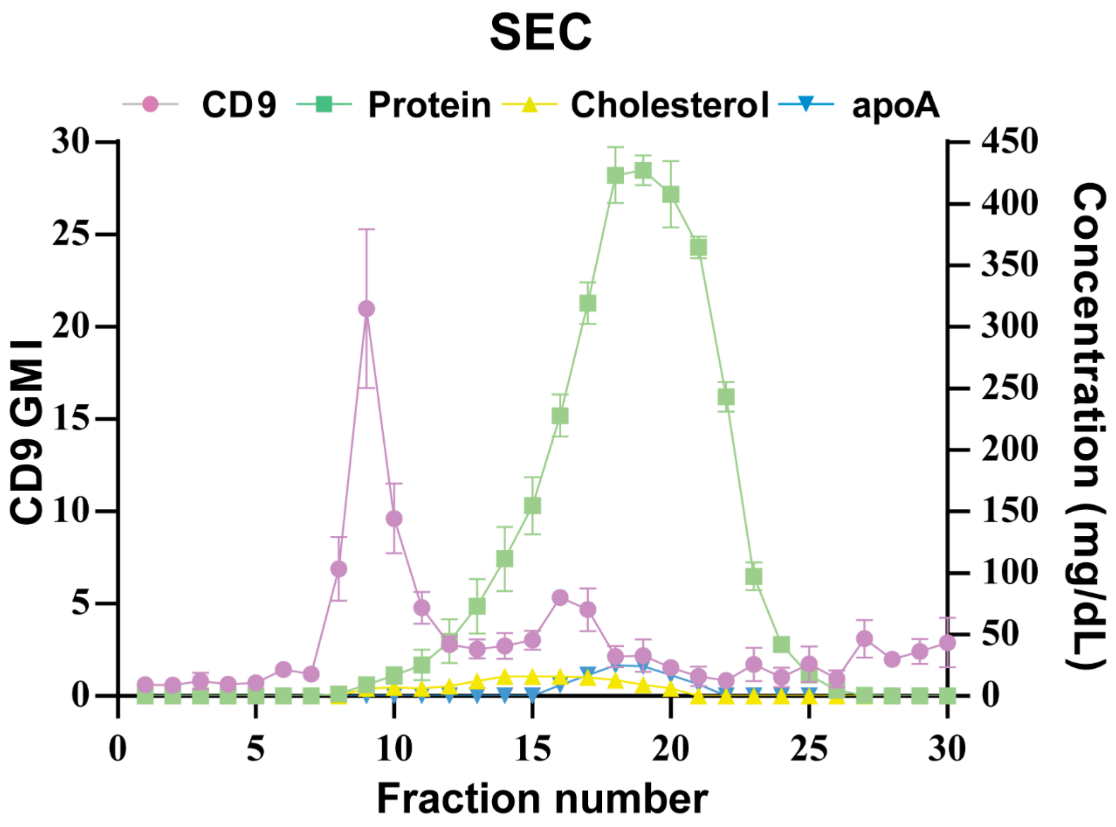

Figure S1. Characterization of all SEC fractions. CD9, ApoA, total cholesterol and total protein quantification. CD9 fluorescence intensity was measured as geometrical mean and plotted on the left ordinate axis. ApoA, total cholesterol and total proteins were measured as mg/dL and plotted on the right ordinate axis, fraction number is given in abscissa.

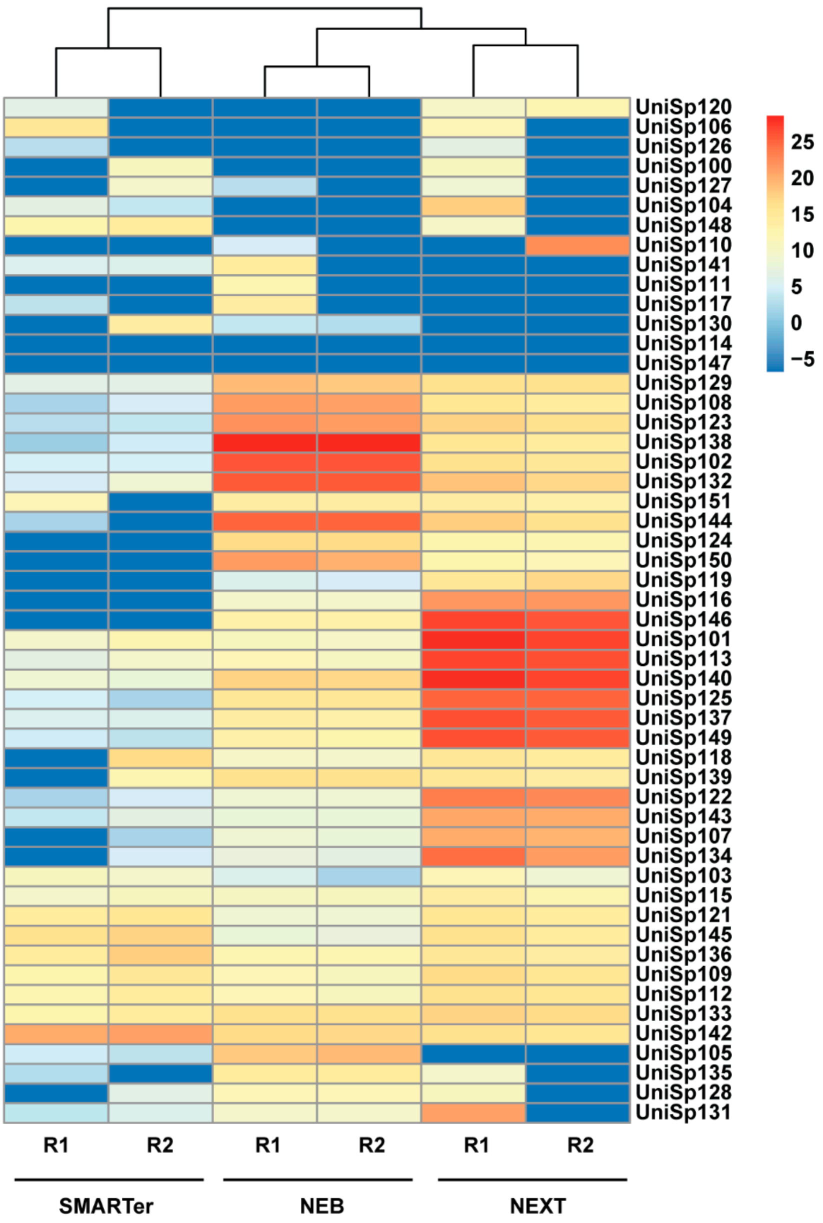

Figure S2. Heat map of normalized NOISeq counts of different spike-ins identified in the two replicas from each protocol (SMARTer, NEB and NEXT). Spike-ins (listed at the right) with a higher number of counts are depicted with red and yellow colors, and those with a lower number of counts are depicted with blue colors (scale at the top right). Heat map was plotted using the "pheatmap" R package over the log2 normalized values.

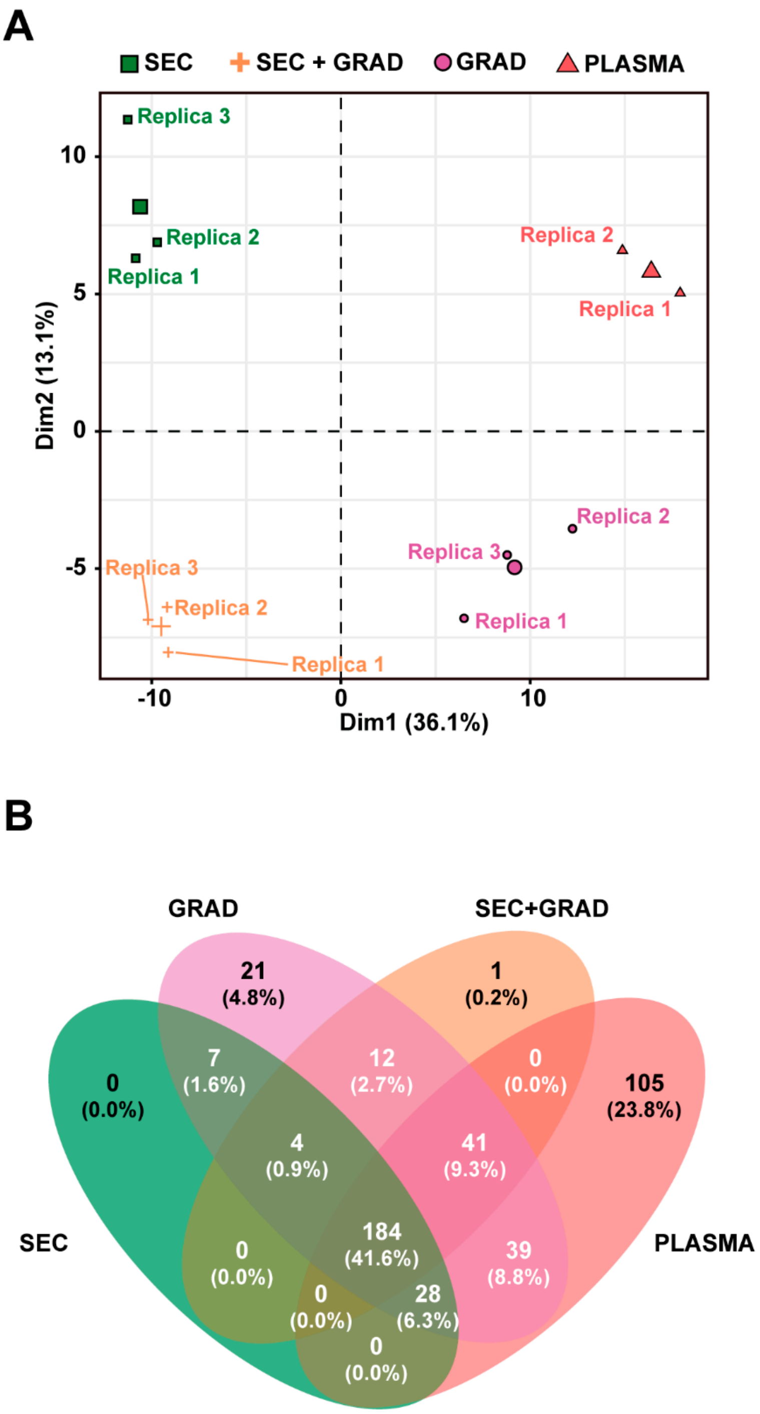

Figure S3. miRNA profiles of different EV isolation methods compared to the plasma sample. (A) Principal component analysis (PCA) showing that miRNA profiles are separated according to the sample. EVs methods were assessed in three independent experiments (Replica 1, Replica 2, and Replica 3). The fourth point is the centroid of the three replicas of each EV isolation method. Plasma sample was assessed in two independent experiments (Replica 1 and Replica 2). The third point is the centroid of the two replicas of each sample. (B) Venn diagram showing the relationship between miRNA profiles of circulating EVs obtained with different EV isolation methods and from the unfractionated plasma sample. Number and percentage of miRNAs detected by one, two, three or four samples are indicated. SEC, Size exclusion chromatography; GRAD, iodixanol gradient; SEC+GRAD, combination of size exclusion chromatography and iodixanol gradient

Table S1. Number of reads that remain after each of the steps of different library preparation methods in the bioinformatics process obtained by the ultra-deep sequencing analysis.

Library preparation method	Replica	Initial reads	Post-trimming[a]	Too short[b]	Too long[c]	Aligned 0 times[d]	Aligned exactly 1 time[e]	Aligned >1 times[f]	No feature[g]	Final reads[h]
**NEBNext Multiplex Small RNA Library Prep Set for Illumina**	**1**	11,892,073	2,701,556	486,910	8,703,607	634,166	785,411	1,281,979	413,514	**1,653,876**
	**2**	12,339,551	2,715,473	631,422	8,992,656	664,003	775,349	1,276,121	402,348	**1,649,122**
**NextFlex Small RNA-Seq v3**	**1**	18,689,055	8,063,509	1,850,873	8,774,673	2,055,648	2,228,291	3,779,570	1,184,404	**4,823,457**
	**2**	11,750,617	5,001,162	1,470,510	5,278,945	1,798,933	1,239,603	1,962,626	682,706	**2,519,523**
**SMARTer smRNA-Seq Kit for Illumina**	**1**	8,738,639	468,249	1,577,605	6,692,785	115,613	61,746	290,890	347,252	**5,384**
	**2**	8,727,581	395,306	1,374,911	6,957,364	91,132	56,476	247,698	285,256	**18,918**

[a] Number of reads between 17 and 25 nucleotides after trimming according to kit manufacturer instructions.
[b] Number of reads discarded to be shorter than 17 nucleotides.
[c] Number of reads discarded to be longer than 25 nucleotides.
[d] Number of reads discarded because do not map to human reference genome (assembly hg38).
[e] Number of reads that map one time to human reference genome (assembly hg38).
[f] Number of reads that map more than one time to human reference genome (assembly hg38).
[g] Number of reads discarded because they do not map to miRNAs coordinates.
[h] Number of reads after each of the steps in the bioinformatics process.

Table S2. Normalized counts of miRNAs detected using three different library preparations, and filtered by abundance (miRNAs with at least 50 reads).

Accession Number[a]	NEBNext[b]		NEXTFlex[c]		SMARTer[d]	
	Replica 1	Replica 2	Replica 1	Replica 2	Replica 1	Replica 2
**MIMAT0000062**	20955.5	19262.1	29495.8	27959.7	22795.2	34786.5
**MIMAT0000062_1**	23376.0	19940.0	29928.9	28755.3	22795.2	37721.2
**MIMAT0000062_2**	22924.3	20133.0	29687.4	29337.8	25210.6	39868.5
**MIMAT0000063**	69745.9	150564.7	43189.6	68745.2	82575.9	94052.4
**MIMAT0000064**	2175.0	2211.6	806.5	1808.9	603.8	2290.5
**MIMAT0000065**	2527.1	1269.6	4470.8	5498.1	4377.9	1717.9
**MIMAT0000066**	1408.3	1493.0	1687.9	1219.6	2113.5	1861.0
**MIMAT0000067**	32428.1	28905.8	26390.8	27383.0	20530.7	33498.1
**MIMAT0000067_1**	45014.8	43239.4	27893.9	28879.4	19021.1	35072.8
**MIMAT0000068**	18.5	66.0	385.2	134.4	0.0	0.0
**MIMAT0000069**	384.5	347.9	12147.5	13791.4	1207.7	2218.9
**MIMAT0000069_1**	370.6	358.0	12187.2	13883.9	3321.2	3221.0
**MIMAT0000070**	433.1	203.1	160.6	0.0	3925.0	787.3
**MIMAT0000072**	4.6	0.0	258.4	0.0	0.0	0.0
**MIMAT0000073**	250.2	71.1	102.5	618.7	6189.4	1789.4
**MIMAT0000074**	169.1	213.3	79.5	120.1	7699.0	3292.5
**MIMAT0000074_1**	159.8	286.9	77.2	116.6	7699.0	3722.0
**MIMAT0000075**	901.0	721.1	486.1	2531.0	301.9	429.5
**MIMAT0000076**	119624.9	103900.0	105219.8	129114.9	2566.3	6871.4
**MIMAT0000077**	8927.0	10669.6	7823.8	9284.3	452.9	429.5
**MIMAT0000078**	440.1	137.1	23312.7	28109.6	30645.2	53038.7
**MIMAT0000079**	2.3	12.7	59.7	0.0	0.0	0.0
**MIMAT0000080**	6992.9	6055.9	13673.0	16933.7	5736.5	10020.8
**MIMAT0000080_1**	6891.0	6388.5	13781.2	16404.0	3472.1	12025.0
**MIMAT0000081**	29713.4	29487.3	4984.8	4458.9	22191.3	17965.9
**MIMAT0000082**	120820.1	106444.2	17445.2	21690.1	301.9	572.6
**MIMAT0000082_1**	116623.0	106444.2	16577.7	20490.1	452.9	143.2
**MIMAT0000083**	4065.1	3382.2	9703.9	9973.6	603.8	143.2
**MIMAT0000084**	16197.8	14529.1	5981.2	6957.8	1811.5	214.7
**MIMAT0000085**	275.6	248.8	820.4	1016.2	0.0	0.0
**MIMAT0000086**	1570.4	1307.7	3734.2	6456.8	2113.5	1861.0
**MIMAT0000087**	3909.9	2861.6	5508.7	4021.2	452.9	0.0
**MIMAT0000088**	491.1	652.6	124.9	64.9	754.8	0.0
**MIMAT0000090**	247.8	162.5	64.1	0.0	0.0	930.5
**MIMAT0000092**	23864.8	24205.8	37890.9	42738.0	8906.7	23548.9
**MIMAT0000092_1**	22449.5	23246.0	35166.6	38624.4	9812.5	24407.8

**MIMAT0000093**	870.9	716.0	4627.0	7978.6	5434.6	5225.1
**MIMAT0000094**	136.7	17.8	48.2	0.0	0.0	0.0
**MIMAT0000095**	57.9	0.0	112.4	168.9	0.0	0.0
**MIMAT0000096**	1556.5	1912.0	2804.8	4320.4	1660.6	715.8
**MIMAT0000097**	28335.2	29616.8	1687.4	2487.9	0.0	0.0
**MIMAT0000098**	6620.0	6441.9	684.5	1.7	0.0	0.0
**MIMAT0000099**	9191.0	9653.9	2550.6	3121.0	905.8	214.7
**MIMAT0000099_1**	9612.6	10568.0	2581.8	2984.8	603.8	429.5
**MIMAT0000100**	30.1	2.5	268.8	454.4	2415.4	3793.6
**MIMAT0000100_1**	18.5	0.0	249.8	420.5	2566.3	3364.1
**MIMAT0000101**	1028.4	774.4	3033.2	2398.9	0.0	0.0
**MIMAT0000101_1**	1109.5	703.3	2875.7	2337.4	0.0	0.0
**MIMAT0000104**	180.7	66.0	320.7	153.4	0.0	0.0
**MIMAT0000222**	12721.1	11489.7	1084.0	748.5	1509.6	357.9
**MIMAT0000227**	379.9	322.5	1823.8	1760.1	754.8	2719.9
**MIMAT0000231**	1894.7	1406.7	388.5	198.2	452.9	0.0
**MIMAT0000231_1**	1836.8	1322.9	397.1	176.4	301.9	0.0
**MIMAT0000232**	322.0	286.9	1885.3	1466.6	0.0	143.2
**MIMAT0000232_1**	296.5	281.8	1879.3	1457.4	452.9	214.7
**MIMAT0000242_1**	85.7	33.0	0.0	0.0	0.0	0.0
**MIMAT0000243**	321547.7	360489.9	3939.9	2659.7	0.0	0.0
**MIMAT0000244**	690.3	553.5	1248.5	960.5	0.0	1073.7
**MIMAT0000244_1**	704.2	604.3	1214.1	888.1	0.0	787.3
**MIMAT0000245**	137636.4	142058.5	52009.5	56214.1	754.8	0.0
**MIMAT0000250**	1362.0	1561.6	806.5	735.3	0.0	357.9
**MIMAT0000252**	854.7	700.8	238.6	426.8	0.0	0.0
**MIMAT0000252_1**	910.3	721.1	238.9	430.8	0.0	0.0
**MIMAT0000252_2**	942.7	713.5	236.0	436.6	0.0	0.0
**MIMAT0000253**	45304.4	55247.1	5628.4	6087.5	0.0	0.0
**MIMAT0000254**	61926.1	77937.0	6771.6	9017.7	0.0	143.2
**MIMAT0000255**	0.0	0.0	65.7	52.3	0.0	0.0
**MIMAT0000256**	3092.3	2303.0	1188.6	1721.1	0.0	0.0
**MIMAT0000256_1**	2964.9	2460.4	1184.7	1727.4	0.0	0.0
**MIMAT0000257**	530.4	335.2	134.8	183.8	0.0	0.0
**MIMAT0000257_1**	460.9	388.5	124.9	170.0	0.0	0.0
**MIMAT0000259**	2652.2	3460.9	1751.3	3446.1	0.0	0.0
**MIMAT0000261**	3020.4	4740.6	619.3	1023.1	1660.6	357.9
**MIMAT0000264**	53.3	0.0	49.8	0.0	0.0	0.0
**MIMAT0000265**	74.1	66.0	0.0	0.0	0.0	0.0
**MIMAT0000271**	20.8	116.8	36.2	0.0	0.0	71.6
**MIMAT0000272**	2413.6	2455.4	904.3	457.8	0.0	0.0

**MIMAT0000278**	5600.8	2595.0	3030.4	1785.4	3170.2	357.9
**MIMAT0000279**	8588.8	8602.7	1646.5	1705.0	603.8	715.8
**MIMAT0000280**	576.8	358.0	12257.6	12464.4	43929.8	67712.0
**MIMAT0000281**	5285.8	5657.3	1551.6	580.8	452.9	143.2
**MIMAT0000318**	111.2	71.1	127.5	0.0	0.0	0.0
**MIMAT0000414**	23174.5	19995.9	39860.7	47158.4	14039.4	12239.7
**MIMAT0000415**	151557.3	144879.5	17704.7	22014.7	8755.8	5511.4
**MIMAT0000416**	416.9	228.5	743.2	931.8	0.0	930.5
**MIMAT0000416_1**	421.6	243.8	653.2	938.7	452.9	1145.2
**MIMAT0000417**	85.7	40.6	3718.0	3270.9	452.9	572.6
**MIMAT0000418**	187.6	78.7	1099.1	1788.3	2868.3	8589.3
**MIMAT0000419**	17754.4	17611.6	10260.1	9037.8	0.0	1145.2
**MIMAT0000420**	60.2	10.2	276.7	248.2	603.8	0.0
**MIMAT0000421**	1314537.7	1453863.0	138996.8	158159.1	29739.4	48171.4
**MIMAT0000423**	664.8	815.1	606.5	514.1	0.0	214.7
**MIMAT0000423_1**	750.5	787.1	618.8	530.8	0.0	143.2
**MIMAT0000424**	24411.4	30089.1	1370.1	2349.5	0.0	71.6
**MIMAT0000424_1**	14859.0	16898.1	927.3	1307.5	0.0	0.0
**MIMAT0000425**	69.5	68.6	37.6	0.0	13737.5	3650.4
**MIMAT0000426**	16.2	63.5	136.6	0.0	0.0	0.0
**MIMAT0000427**	0.0	0.0	0.0	84.4	0.0	0.0
**MIMAT0000427_1**	0.0	0.0	0.0	85.6	0.0	0.0
**MIMAT0000431**	101.9	86.3	145.5	0.0	0.0	0.0
**MIMAT0000432**	4.6	0.0	47.7	0.0	0.0	0.0
**MIMAT0000433**	10416.4	11238.3	6038.6	9689.3	15549.0	5654.6
**MIMAT0000434**	555.9	464.7	341.9	449.2	7850.0	3364.1
**MIMAT0000435**	23105.0	23378.1	11082.8	11873.3	301.9	572.6
**MIMAT0000436**	945.0	799.8	1080.1	1033.4	10869.2	715.8
**MIMAT0000437**	0.0	0.0	289.2	0.0	754.8	787.3
**MIMAT0000438**	1948.0	1223.9	190.4	175.2	0.0	0.0
**MIMAT0000440**	8584.2	7391.5	15001.9	15461.9	18568.3	12382.8
**MIMAT0000442**	2.3	0.0	0.0	182.7	0.0	0.0
**MIMAT0000442_1**	2.3	0.0	0.0	168.9	0.0	0.0
**MIMAT0000442_2**	4.6	0.0	0.0	170.6	0.0	0.0
**MIMAT0000443**	5327.5	8389.4	3354.8	2528.2	905.8	1503.1
**MIMAT0000444**	1714.1	1868.8	3805.4	2856.7	74273.0	287597.0
**MIMAT0000445**	48412.8	45631.3	12224.7	20177.6	9661.5	6298.8
**MIMAT0000446**	528.1	840.5	0.0	0.0	0.0	0.0
**MIMAT0000447**	6529.6	5383.0	476.7	161.4	301.9	787.3
**MIMAT0000449**	23702.6	18645.1	28003.7	27081.9	18568.3	23405.7
**MIMAT0000451**	956.6	441.8	3308.1	4322.2	17813.4	53682.9

**MIMAT0000453**	0.0	0.0	98.6	0.0	0.0	0.0
**MIMAT0000454**	27.8	233.6	0.0	0.0	0.0	0.0
**MIMAT0000455**	5735.1	2983.5	3435.9	6293.7	3321.2	1145.2
**MIMAT0000456**	2717.0	1404.2	2935.7	2654.0	2264.4	1646.3
**MIMAT0000460**	595.3	586.5	312.4	413.0	151.0	214.7
**MIMAT0000460_1**	484.1	520.5	558.0	411.3	452.9	0.0
**MIMAT0000461**	25.5	58.4	64.9	0.0	151.0	0.0
**MIMAT0000462**	157.5	210.8	0.0	197.6	0.0	357.9
**MIMAT0000510**	46837.8	21384.8	14197.4	8556.5	151.0	858.9
**MIMAT0000617**	127.4	101.6	137.2	0.0	0.0	0.0
**MIMAT0000646**	741.2	530.7	332.0	373.4	0.0	0.0
**MIMAT0000680**	118.1	119.3	625.3	348.7	0.0	0.0
**MIMAT0000681**	32.4	96.5	518.4	619.8	1207.7	858.9
**MIMAT0000682**	539.7	248.8	0.0	0.0	0.0	0.0
**MIMAT0000688**	0.0	0.0	25.3	0.0	603.8	143.2
**MIMAT0000689**	5390.0	6459.6	789.1	524.5	0.0	0.0
**MIMAT0000691**	30.1	35.5	147.1	0.0	603.8	357.9
**MIMAT0000692**	11498.1	6881.1	19926.9	26585.6	0.0	71.6
**MIMAT0000693**	3495.3	3839.2	540.0	0.0	1962.5	357.9
**MIMAT0000703**	88.0	116.8	691.3	842.1	151.0	0.0
**MIMAT0000707**	2358.0	2440.1	668.6	923.7	151.0	0.0
**MIMAT0000720**	4.6	0.0	20.1	0.0	0.0	858.9
**MIMAT0000721**	115.8	30.5	1896.6	2228.9	0.0	214.7
**MIMAT0000722**	148.2	119.3	0.0	158.0	0.0	0.0
**MIMAT0000727**	169.1	157.4	597.2	492.9	603.8	0.0
**MIMAT0000728**	3233.5	4273.4	885.6	968.5	301.9	0.0
**MIMAT0000729**	9.3	0.0	103.5	0.0	151.0	429.5
**MIMAT0000729_1**	30.1	0.0	87.9	0.0	0.0	214.7
**MIMAT0000732**	2812.0	2800.7	77.2	143.0	301.9	0.0
**MIMAT0000733**	593.0	454.5	99.6	384.9	0.0	0.0
**MIMAT0000736**	1637.6	1505.7	37.3	181.5	0.0	0.0
**MIMAT0000737**	3534.7	3813.8	2760.5	4922.5	1811.5	1503.1
**MIMAT0000750**	335.9	269.2	179.9	446.3	754.8	787.3
**MIMAT0000751**	1040.0	601.8	259.5	283.2	0.0	143.2
**MIMAT0000752**	3604.2	4288.6	820.9	1495.3	1962.5	3077.8
**MIMAT0000753**	150.6	0.0	1511.9	1573.4	5283.6	6585.1
**MIMAT0000754**	11.6	0.0	33.6	116.0	151.0	214.7
**MIMAT0000755**	108.9	33.0	133.5	0.0	0.0	0.0
**MIMAT0000756**	57.9	2.5	147.1	209.1	1358.7	572.6
**MIMAT0000757**	78473.7	86443.2	4832.3	5943.8	2717.3	10020.8
**MIMAT0000759**	8164.9	7840.9	1579.2	2203.0	151.0	286.3

**MIMAT0000760**	60.2	0.0	188.8	0.0	0.0	71.6
**MIMAT0000762**	0.0	0.0	25.6	0.0	0.0	0.0
**MIMAT0000764**	55.6	96.5	103.0	0.0	0.0	0.0
**MIMAT0000765**	233.9	246.3	7986.7	9940.9	2566.3	3507.3
**MIMAT0000772**	67.2	142.2	170.3	418.8	151.0	71.6
**MIMAT0001340**	17416.2	19731.8	7633.9	10677.9	301.9	501.0
**MIMAT0001343**	210.8	134.6	62.8	0.0	0.0	0.0
**MIMAT0001536**	48.6	20.3	27.1	0.0	0.0	0.0
**MIMAT0001545**	37.1	0.0	39.1	0.0	0.0	0.0
**MIMAT0001545_1**	32.4	0.0	40.9	0.0	0.0	0.0
**MIMAT0001618**	71.8	71.1	123.3	0.0	301.9	143.2
**MIMAT0001621**	192.3	228.5	131.2	0.0	0.0	0.0
**MIMAT0001629**	6.9	0.0	116.6	0.0	0.0	0.0
**MIMAT0001629_1**	2.3	0.0	117.6	0.0	0.0	0.0
**MIMAT0001630**	9.3	0.0	0.3	553.2	0.0	0.0
**MIMAT0001631**	38927.6	35853.0	36311.1	47680.0	6642.3	2648.4
**MIMAT0001635**	85.7	86.3	276.2	0.0	0.0	0.0
**MIMAT0001636**	30.1	0.0	44.9	0.0	0.0	0.0
**MIMAT0001639**	34512.8	40431.1	4898.0	5745.1	2113.5	4366.2
**MIMAT0002171**	104.2	0.0	38.1	0.0	0.0	0.0
**MIMAT0002173**	37.1	109.2	285.0	107.4	1207.7	3149.4
**MIMAT0002174**	1107.2	1376.2	2414.4	819.2	452.9	1431.5
**MIMAT0002175**	2388.1	2419.8	531.7	318.2	0.0	0.0
**MIMAT0002176**	958.9	2920.0	1252.2	1777.4	301.9	214.7
**MIMAT0002177**	57302.8	83477.5	28584.1	27261.2	4679.8	13528.1
**MIMAT0002177_1**	63362.2	92697.2	28600.0	27322.6	6642.3	13098.6
**MIMAT0002178**	48.6	0.0	88.7	526.2	0.0	0.0
**MIMAT0002808**	289.5	134.6	0.0	0.0	452.9	0.0
**MIMAT0002809**	14861.3	13480.4	1445.4	1236.8	1962.5	3507.3
**MIMAT0002813**	90.3	48.2	84.2	0.0	0.0	0.0
**MIMAT0002814**	213.1	157.4	71.2	0.0	0.0	0.0
**MIMAT0002816**	25.5	0.0	451.9	868.6	0.0	0.0
**MIMAT0002817**	755.1	634.8	502.2	1647.5	0.0	143.2
**MIMAT0002818**	23.2	0.0	21.6	0.0	0.0	143.2
**MIMAT0002821**	227.0	86.3	0.0	0.0	0.0	0.0
**MIMAT0002859**	30.1	157.4	0.0	0.0	0.0	0.0
**MIMAT0002859_1**	30.1	129.5	0.0	0.0	0.0	0.0
**MIMAT0002870**	178.4	137.1	115.3	172.3	0.0	0.0
**MIMAT0002871**	55.6	10.2	165.6	123.5	0.0	0.0
**MIMAT0002874**	18.5	129.5	0.0	199.9	0.0	0.0
**MIMAT0002875**	0.0	0.0	47.7	0.0	0.0	0.0

**MIMAT0002876**	30.1	0.0	216.4	87.3	452.9	0.0
**MIMAT0002888**	3770.9	3478.7	32.9	66.6	0.0	143.2
**MIMAT0002891**	30.1	0.0	52.4	196.5	0.0	0.0
**MIMAT0003161**	39.4	139.7	0.0	0.0	0.0	0.0
**MIMAT0003180**	6.9	0.0	1147.9	841.0	0.0	357.9
**MIMAT0003218**	484.1	942.0	388.3	0.0	0.0	357.9
**MIMAT0003220**	11.6	0.0	103.0	455.0	0.0	0.0
**MIMAT0003239**	0.0	58.4	578.1	701.4	1962.5	3292.5
**MIMAT0003241**	39.4	0.0	87.4	0.0	452.9	0.0
**MIMAT0003249**	6965.1	5504.9	1456.9	871.4	0.0	0.0
**MIMAT0003251**	0.0	0.0	0.0	54.0	0.0	0.0
**MIMAT0003251_1**	0.0	0.0	0.0	54.0	0.0	0.0
**MIMAT0003251_2**	0.0	0.0	0.0	58.0	0.0	0.0
**MIMAT0003266**	30.1	0.0	118.1	0.0	0.0	0.0
**MIMAT0003283**	74.1	96.5	0.0	0.0	0.0	0.0
**MIMAT0003297**	416.9	698.3	242.0	198.8	301.9	429.5
**MIMAT0003321**	92.7	43.2	72.8	0.0	0.0	0.0
**MIMAT0003322**	134.3	149.8	3307.3	3786.8	0.0	0.0
**MIMAT0003329**	111.2	154.9	1.3	0.0	0.0	0.0
**MIMAT0003330**	509.6	540.8	0.0	0.0	0.0	0.0
**MIMAT0003332**	44.0	0.0	175.5	286.7	0.0	1073.7
**MIMAT0003338**	53.3	193.0	178.1	0.0	151.0	501.0
**MIMAT0003339**	148.2	231.1	347.3	691.1	0.0	1288.4
**MIMAT0003389**	20.8	63.5	56.8	0.0	0.0	0.0
**MIMAT0003393**	549.0	479.9	2585.2	2874.6	603.8	787.3
**MIMAT0003884**	60.2	45.7	52.2	0.0	0.0	71.6
**MIMAT0003885**	11.6	0.0	74.6	0.0	151.0	0.0
**MIMAT0003886**	111.2	124.4	0.0	0.0	0.0	0.0
**MIMAT0003888**	9.3	0.0	65.5	0.0	0.0	0.0
**MIMAT0003945**	0.0	0.0	0.0	236.1	0.0	0.0
**MIMAT0004481**	521.2	578.9	48.2	0.0	603.8	572.6
**MIMAT0004481_1**	437.8	571.3	48.0	0.0	905.8	1360.0
**MIMAT0004482**	199.2	766.8	512.1	272.3	2415.4	9018.7
**MIMAT0004484**	11822.4	25381.5	13493.6	16784.9	19172.1	130485.1
**MIMAT0004485**	11.6	116.8	0.0	0.0	0.0	0.0
**MIMAT0004486**	48.6	109.2	47.7	0.0	452.9	1717.9
**MIMAT0004494**	20.8	0.0	114.7	184.4	0.0	143.2
**MIMAT0004495**	393.8	386.0	18.3	725.5	151.0	0.0
**MIMAT0004496**	9.3	83.8	123.6	0.0	0.0	357.9
**MIMAT0004497**	120.4	167.6	0.0	0.0	0.0	286.3
**MIMAT0004498**	85.7	38.1	0.0	287.8	0.0	0.0

**MIMAT0004500**	48.6	114.3	40.4	0.0	301.9	572.6
**MIMAT0004501**	108.9	116.8	0.0	0.0	0.0	0.0
**MIMAT0004502**	5529.0	5085.9	1422.0	263.7	603.8	286.3
**MIMAT0004518**	4486.7	4933.6	1241.8	1964.0	0.0	143.2
**MIMAT0004549**	233.9	286.9	0.0	0.0	0.0	0.0
**MIMAT0004551**	90.3	76.2	0.0	0.0	0.0	0.0
**MIMAT0004552**	845.4	1254.3	108.7	0.0	0.0	0.0
**MIMAT0004556**	104.2	81.3	44.3	0.0	2113.5	2863.1
**MIMAT0004558**	356.7	586.5	14.6	640.5	0.0	0.0
**MIMAT0004563**	322.0	233.6	1897.3	1487.3	151.0	143.2
**MIMAT0004568**	90.3	147.3	40.9	0.0	0.0	0.0
**MIMAT0004570**	5464.1	4303.9	162.5	1552.7	603.8	1073.7
**MIMAT0004585**	18.5	0.0	39.1	0.0	603.8	1789.4
**MIMAT0004586**	92.7	38.1	268.6	360.8	1358.7	858.9
**MIMAT0004587**	53.3	0.0	97.8	0.0	0.0	0.0
**MIMAT0004589**	48.6	121.9	0.0	0.0	0.0	0.0
**MIMAT0004597**	6881.7	6274.3	1398.0	1544.7	452.9	71.6
**MIMAT0004600**	690.3	289.5	2382.4	525.0	1056.7	858.9
**MIMAT0004601**	535.1	434.2	320.0	467.6	0.0	143.2
**MIMAT0004602**	118.1	30.5	0.0	0.0	0.0	0.0
**MIMAT0004603**	44.0	127.0	0.0	0.0	0.0	0.0
**MIMAT0004606**	20.8	0.0	43.0	0.0	0.0	0.0
**MIMAT0004608**	71.8	104.1	0.0	0.0	0.0	0.0
**MIMAT0004610**	525.8	515.4	102.0	194.7	0.0	715.8
**MIMAT0004611**	136.7	53.3	0.0	0.0	151.0	0.0
**MIMAT0004614**	829.2	782.1	462.1	0.0	0.0	0.0
**MIMAT0004672**	1535.7	1371.1	284.5	0.0	754.8	0.0
**MIMAT0004674**	27.8	0.0	21.4	0.0	0.0	0.0
**MIMAT0004678**	108.9	40.6	0.0	0.0	0.0	0.0
**MIMAT0004680**	945.0	1183.2	530.1	402.7	1207.7	858.9
**MIMAT0004682**	1711.7	1152.8	61.3	868.6	452.9	0.0
**MIMAT0004683**	0.0	0.0	58.9	377.4	151.0	0.0
**MIMAT0004688**	78.8	127.0	0.0	0.0	0.0	0.0
**MIMAT0004690**	41.7	0.0	16.9	0.0	0.0	0.0
**MIMAT0004692**	7710.9	8244.7	2077.0	4641.0	0.0	0.0
**MIMAT0004694**	1239.2	1241.7	119.4	0.0	0.0	787.3
**MIMAT0004697**	46.3	2.5	1627.2	1814.7	0.0	286.3
**MIMAT0004699**	53.3	0.0	93.1	0.0	0.0	0.0
**MIMAT0004701**	229.3	304.7	187.8	0.0	0.0	0.0
**MIMAT0004702**	426.2	302.2	708.5	329.7	0.0	0.0
**MIMAT0004703**	993.7	1069.0	474.1	1086.3	0.0	0.0

**MIMAT0004748**	211764.7	287940.9	42571.3	57079.8	23248.1	11237.6
**MIMAT0004749**	252.5	274.2	210.7	0.0	0.0	0.0
**MIMAT0004761**	1176.7	3374.5	1153.9	2370.2	301.9	0.0
**MIMAT0004762**	354.4	391.0	362.5	572.7	0.0	0.0
**MIMAT0004762_1**	257.1	284.4	236.3	550.9	0.0	0.0
**MIMAT0004766**	25.5	38.1	75.4	0.0	0.0	0.0
**MIMAT0004767**	201.5	639.9	0.0	380.9	905.8	0.0
**MIMAT0004774**	542.0	624.6	448.8	440.6	0.0	0.0
**MIMAT0004775**	51.0	43.2	388.8	112.0	0.0	0.0
**MIMAT0004776**	125.1	27.9	0.0	0.0	452.9	0.0
**MIMAT0004780**	4.6	0.0	100.1	0.0	0.0	143.2
**MIMAT0004792**	90.3	144.7	0.0	0.0	0.0	0.0
**MIMAT0004793**	0.0	0.0	0.0	0.0	2868.3	3936.7
**MIMAT0004795**	0.0	0.0	25.0	0.0	0.0	0.0
**MIMAT0004796**	41.7	68.6	32.3	0.0	0.0	0.0
**MIMAT0004797**	37.1	139.7	0.0	0.0	0.0	0.0
**MIMAT0004799**	108.9	35.5	0.0	0.0	0.0	0.0
**MIMAT0004801**	34.7	0.0	0.0	63.2	0.0	644.2
**MIMAT0004808**	1223.0	1279.7	774.5	704.9	2264.4	2004.2
**MIMAT0004809**	4.6	27.9	125.2	0.0	0.0	143.2
**MIMAT0004810**	1690.9	1399.1	183.6	223.5	452.9	71.6
**MIMAT0004813**	78.8	177.7	52.9	0.0	0.0	0.0
**MIMAT0004814**	555.9	299.6	55.0	418.8	0.0	71.6
**MIMAT0004819**	731.9	840.5	31.3	0.0	0.0	0.0
**MIMAT0004909**	44.0	63.5	120.0	0.6	0.0	0.0
**MIMAT0004921**	308.1	477.4	59.2	49.4	0.0	0.0
**MIMAT0004945**	7852.2	7053.8	236.3	970.8	1056.7	286.3
**MIMAT0004947**	34.7	175.2	970.6	726.1	0.0	2863.1
**MIMAT0004948**	60.2	33.0	42.2	0.0	0.0	0.0
**MIMAT0004949**	37.1	73.6	87.4	195.9	301.9	71.6
**MIMAT0004954**	10448.8	13673.4	1012.6	1013.3	301.9	286.3
**MIMAT0004955**	48.6	134.6	255.6	0.0	1056.7	3221.0
**MIMAT0004957**	264.1	307.2	77.7	463.0	0.0	0.0
**MIMAT0004959**	18.5	0.0	41.2	0.0	0.0	0.0
**MIMAT0004984**	134.3	180.3	0.0	0.0	0.0	0.0
**MIMAT0004984_1**	148.2	167.6	0.0	0.0	0.0	0.0
**MIMAT0004984_2**	127.4	180.3	0.0	0.0	0.0	0.0
**MIMAT0004984_3**	143.6	167.6	0.0	0.0	0.0	0.0
**MIMAT0004984_4**	180.7	180.3	0.0	0.0	0.0	0.0
**MIMAT0004985**	115.8	294.5	265.7	0.0	0.0	0.0
**MIMAT0005577**	0.0	0.0	43.5	0.0	0.0	0.0

**MIMAT0005582**	97.3	116.8	0.0	0.0	0.0	0.0
**MIMAT0005583**	0.0	0.0	0.0	159.1	0.0	0.0
**MIMAT0005584**	0.0	99.0	0.0	0.0	754.8	1861.0
**MIMAT0005593**	11.6	0.0	36.0	0.0	0.0	0.0
**MIMAT0005792**	1857.7	1277.2	340.8	264.2	0.0	71.6
**MIMAT0005792_1**	1697.8	1081.7	347.9	279.8	151.0	71.6
**MIMAT0005793**	711.1	617.0	166.9	94.2	0.0	0.0
**MIMAT0005793_1**	715.7	540.8	207.6	96.5	0.0	0.0
**MIMAT0005797**	576.8	812.5	304.3	23.0	151.0	0.0
**MIMAT0005825**	201.5	134.6	0.0	0.0	0.0	0.0
**MIMAT0005874**	48.6	99.0	78.8	0.0	0.0	0.0
**MIMAT0005875**	51.0	81.3	0.0	258.5	0.0	0.0
**MIMAT0005882**	20.8	0.0	92.6	0.0	0.0	0.0
**MIMAT0005884**	27.8	0.0	0.0	164.9	0.0	0.0
**MIMAT0005892**	46.3	50.8	143.4	0.0	0.0	0.0
**MIMAT0005898**	120.4	121.9	0.0	0.0	1207.7	644.2
**MIMAT0005901**	13.9	22.9	366.9	649.1	0.0	0.0
**MIMAT0005911**	16.2	38.1	633.4	1223.6	603.8	357.9
**MIMAT0005919**	90.3	121.9	0.0	0.0	0.0	0.0
**MIMAT0005919_1**	85.7	129.5	0.0	0.0	0.0	0.0
**MIMAT0005933**	0.0	0.0	88.4	0.0	0.0	0.0
**MIMAT0005948**	613.8	589.1	418.5	0.0	301.9	0.0
**MIMAT0005949**	0.0	0.0	197.9	0.0	2717.3	4079.9
**MIMAT0005950**	9.3	7.6	91.3	0.0	0.0	0.0
**MIMAT0005951**	3493.0	3143.5	130.1	0.0	0.0	0.0
**MIMAT0006764**	150.6	157.4	89.2	48.8	0.0	0.0
**MIMAT0006764_1**	122.8	114.3	84.5	36.8	0.0	0.0
**MIMAT0006789**	206.2	114.3	37.8	0.0	301.9	0.0
**MIMAT0007881**	275.6	370.7	120.2	0.0	0.0	143.2
**MIMAT0007883**	0.0	0.0	28.9	0.0	0.0	0.0
**MIMAT0010133**	423.9	246.3	100.1	0.0	0.0	0.0
**MIMAT0010214**	9.3	15.2	92.3	68.4	0.0	0.0
**MIMAT0011161**	0.0	0.0	43.8	0.0	0.0	0.0
**MIMAT0011778**	0.0	0.0	18.0	0.0	0.0	0.0
**MIMAT0014982**	150.6	55.9	55.5	0.0	0.0	0.0
**MIMAT0015004**	23.2	0.0	0.3	339.5	0.0	0.0
**MIMAT0015006**	162.1	317.4	0.0	0.0	0.0	0.0
**MIMAT0015032**	104.2	53.3	0.0	0.0	0.0	0.0
**MIMAT0015032_1**	141.3	53.3	0.0	0.0	0.0	0.0
**MIMAT0015041**	16.2	33.0	1235.0	1826.2	5585.6	3650.4
**MIMAT0015043**	90.3	601.8	0.0	0.0	0.0	0.0

**MIMAT0015050**	2290.8	2823.5	782.8	782.4	754.8	429.5
**MIMAT0015054**	32.4	129.5	92.3	0.0	0.0	0.0
**MIMAT0016847**	71.8	53.3	2.1	0.0	0.0	0.0
**MIMAT0016916**	0.0	0.0	35.7	0.0	0.0	0.0
**MIMAT0017981**	57.9	104.1	0.0	0.0	0.0	0.0
**MIMAT0017982**	104.2	195.5	0.0	0.0	0.0	143.2
**MIMAT0017988**	16.2	0.0	38.9	0.0	0.0	0.0
**MIMAT0017990**	69.5	73.6	1298.4	1597.0	0.0	0.0
**MIMAT0017994**	2230.6	2272.5	322.6	273.4	0.0	143.2
**MIMAT0017997**	13.9	0.0	24.0	0.6	0.0	0.0
**MIMAT0018104**	39.4	307.2	0.0	367.6	0.0	0.0
**MIMAT0018195**	2.3	0.0	24.8	0.0	0.0	0.0
**MIMAT0018205**	18.5	0.0	96.5	0.0	0.0	0.0
**MIMAT0018356**	0.0	0.0	0.3	253.9	0.0	0.0
**MIMAT0018926**	11.6	33.0	31.0	0.0	0.0	0.0
**MIMAT0018926_1**	27.8	20.3	39.1	0.0	0.0	0.0
**MIMAT0018965**	1285.5	952.2	351.5	465.3	301.9	71.6
**MIMAT0019048**	0.0	60.9	77.7	0.0	0.0	0.0
**MIMAT0019074**	104.2	78.7	0.3	0.0	0.0	0.0
**MIMAT0019198**	39.4	86.3	0.0	0.0	0.0	0.0
**MIMAT0019214**	13.9	0.0	66.5	0.0	0.0	0.0
**MIMAT0019734**	44.0	0.0	71.7	0.0	0.0	0.0
**MIMAT0019739**	78.8	93.9	225.0	0.0	0.0	0.0
**MIMAT0019772**	0.0	96.5	20.1	0.0	0.0	0.0
**MIMAT0019855**	69.5	25.4	0.0	442.9	0.0	0.0
**MIMAT0019856**	25.5	0.0	63.1	0.0	0.0	0.0
**MIMAT0019880**	99.6	116.8	0.0	0.0	0.0	0.0
**MIMAT0019892**	11.6	0.0	51.4	0.0	0.0	0.0
**MIMAT0020924**	55.6	71.1	0.0	0.0	0.0	143.2
**MIMAT0021120**	44.0	104.1	0.0	1.7	0.0	0.0
**MIMAT0022697**	220.0	162.5	616.5	1001.8	0.0	429.5
**MIMAT0022700**	0.0	0.0	92.1	0.0	0.0	0.0
**MIMAT0022714**	141.3	190.4	0.0	0.0	452.9	0.0
**MIMAT0022720**	57.9	50.8	87.4	0.0	0.0	0.0
**MIMAT0022726**	41.7	0.0	307.7	356.7	452.9	1360.0
**MIMAT0022727**	132.0	22.9	98.8	0.0	0.0	0.0
**MIMAT0022842**	27.8	0.0	54.0	227.5	3623.1	9090.3
**MIMAT0025479**	0.0	0.0	3.1	33.9	0.0	286.3
**MIMAT0025479_1**	0.0	0.0	2.9	36.8	0.0	214.7
**MIMAT0025479_2**	0.0	0.0	5.0	29.9	0.0	214.7
**MIMAT0025479_3**	0.0	0.0	8.3	50.6	0.0	357.9

**MIMAT0025483**	18.5	48.2	73.0	0.0	0.0	0.0
**MIMAT0025848**	0.0	0.0	15.6	137.9	0.0	71.6
**MIMAT0025848_1**	0.0	0.0	13.3	121.8	0.0	214.7
**MIMAT0026555**	0.0	0.0	44.3	0.0	0.0	0.0
**MIMAT0026555_1**	0.0	0.0	55.5	0.0	0.0	0.0
**MIMAT0026559**	171.4	91.4	91.3	0.0	0.0	0.0
**MIMAT0026606**	4.6	0.0	24.0	0.0	0.0	0.0
**MIMAT0026721**	0.0	0.0	38.3	0.0	0.0	0.0
**MIMAT0027032**	2.3	2.5	58.7	0.6	0.0	0.0
**MIMAT0027366**	0.0	0.0	0.0	125.2	0.0	0.0
**MIMAT0027369**	2.3	60.9	8.9	0.0	0.0	0.0
**MIMAT0027384**	37.1	121.9	98.3	0.0	0.0	0.0
**MIMAT0027395**	60.2	142.2	0.0	0.0	0.0	0.0
**MIMAT0027507**	0.0	0.0	449.8	221.7	0.0	0.0
**MIMAT0027518**	0.0	0.0	97.3	0.0	0.0	357.9
**MIMAT0027530**	11.6	165.0	0.0	0.0	0.0	0.0
**MIMAT0027539**	0.0	0.0	62.8	0.0	0.0	0.0
**MIMAT0027587**	630.0	637.3	87.1	0.0	0.0	0.0
**MIMAT0027588**	37.1	93.9	42.5	0.0	0.0	0.0
**MIMAT0027604**	143.6	40.6	69.4	0.0	0.0	0.0
**MIMAT0027617**	0.0	0.0	25.8	0.0	0.0	0.0
**MIMAT0030021**	155.2	292.0	0.0	0.0	0.0	0.0
**MIMAT0030413**	637.0	561.2	17060.3	24216.0	754.8	3722.0
**MIMAT0030414**	3015.8	10232.8	1051.4	631.3	2566.3	3865.2
**MIMAT0030428**	0.0	0.0	34.9	0.0	0.0	0.0
**MIMAT0030999**	0.0	0.0	79.5	0.0	0.0	0.0
**MIMAT0031180**	0.0	0.0	12.5	23.0	0.0	0.0
**MIMAT0039763**	9.3	20.3	14.9	0.6	0.0	0.0
**MIMAT0039764**	616.1	1000.4	117.1	0.0	0.0	0.0
**MIMAT0041618**	0.0	0.0	42.5	0.0	0.0	0.0
**MIMAT0041629**	53.3	38.1	34.9	259.7	0.0	0.0
**MIMAT0041630**	57.9	246.3	25.0	0.0	0.0	0.0
**MIMAT0041997**	20.8	0.0	28.7	0.0	0.0	0.0
**MIMAT0044657**	129.7	190.4	0.0	0.0	0.0	0.0
**MIMAT0044658**	208.5	162.5	0.0	0.0	0.0	0.0
**MIMAT0049032**	0.0	0.0	102.2	0.6	24606.7	30778.2

[a] Accession number of miRNAs in miRbase (the microRNA database) (https://www.mirbase.org/index.shtml)

[b] NEBNext Multiplex Small RNA Library Prep Set for Illumina.

[c] NEXTFlex Small RNA-Seq Kit v3.

[d] SMARTer smRNA-seq kit.

Table S3. Number of reads that remain after each of the steps of different EVs isolation methods in the bioinformatics process obtained by the ultra-deep sequencing analysis.

Isolation methods	Replica	Initial reads	Post-trimming[a]	Too short[b]	Too long[c]	Aligned 0 times[d]	Aligned exactly 1 time[e]	Aligned >1 times[f]	No feature[g]	Final reads[h]
Size exclusion chromatography (SEC)	1	52,212,398	14,869,057	15,021,795	22,321,546	6,518,235	643,691	7,707,131	8,120,100	**230,722**
	2	11,845,277	2,866,224	6,138,591	2,840,462	1,962,382	217,196	686,646	870,412	**33,430**
	3	13,457,256	4,050,107	7,021,869	2,385,280	2,612,502	329,589	1,108,016	1,341,088	**96,517**
Iodixanol gradients	1	22,927,102	5,503,171	14,513,590	2,910,341	2,661,171	763,814	2,078,186	1,831,390	**1,010,610**
	2	19,791,596	7,534,761	8,199,092	4,057,743	3,536,190	1,195,765	2,802,806	1,979,942	**2,018,629**
	3	14,796,045	5,223,574	6,714,444	2,858,027	2,509,192	812,122	1,902,260	1,484,901	**1,229,481**
SEC + Iodixanol gradients	1	20,277,248	7,604,405	8,439,115	4,233,728	5,101,523	480,885	2,021,997	2,383,242	**119,640**
	2	17,261,430	5,452,052	8,571,717	3,237,661	3,726,289	391,554	1,334,209	1,662,167	**63,596**
	3	22,873,867	7,761,546	12,309,751	2,802,570	5,108,336	650,194	2,003,016	2,523,274	**129,936**

[a] Number of reads between 17 and 25 nucleotides after trimming according to kit manufacturer instructions.
[b] Number of reads discarded to be shorter than 17 nucleotides.
[c] Number of reads discarded to be longer than 25 nucleotides.
[d] Number of reads discarded because do not map to human reference genome (assembly hg38).
[e] Number of reads that map one time to human reference genome (assembly hg38).
[f] Number of reads that map more than one time to human reference genome (assembly hg38).
[g] Number of reads discarded because they do not map to miRNAs coordinates.
[h] Number of reads after each of the steps in the bioinformatics process.

Table S4. Normalized counts of miRNAs detected using three different EVs isolation methods and filtered by abundance (miRNAs with at least 50 reads).

Accession Number[a]	SEC[b]			GRAD[c]			SEC + GRAD[d]		
	R1	R2	R3	R1	R2	R3	R1	R2	R3
**MIMAT0000062**	15754,4	6086,6	4868,5	10488,2	10738,7	10522,4	9542,1	9454,2	10963,7
**MIMAT0000062_1**	16424,7	6486,6	4956,3	10949,6	11003,6	10795,8	10158,6	10181,5	11847,9
**MIMAT0000062_2**	16151,2	6186,6	5229,1	10933,0	11042,5	10870,5	10132,6	10058,9	11725,0
**MIMAT0000063**	189016,8	42643,9	59573,3	75981,4	78065,5	71599,1	76615,0	74547,1	67494,9
**MIMAT0000064**	4091,2	662,4	1488,8	1406,0	1563,3	1534,5	1528,1	1487,2	2125,3
**MIMAT0000065**	450,9	62,5	887,7	670,4	742,6	1077,5	655,5	1168,5	1011,1
**MIMAT0000066**	998,0	562,4	1382,4	282,6	327,4	327,5	138,9	416,7	392,6
**MIMAT0000067**	15378,7	7886,4	7901,5	14882,1	15146,2	14775,2	14981,8	13025,1	13743,2
**MIMAT0000067_1**	19731,3	10648,5	11540,2	18609,1	18973,3	19004,8	15619,9	14994,4	16225,4
**MIMAT0000069**	90,2	225,0	0,0	234,5	214,7	246,6	464,5	392,2	130,9
**MIMAT0000069_1**	78,2	200,0	4,6	239,9	194,0	236,8	468,9	424,9	91,2
**MIMAT0000070**	336,7	12,5	13,9	233,3	197,9	221,3	4,3	0,0	646,3
**MIMAT0000072**	0,0	0,0	0,0	17,2	40,2	17,5	0,0	0,0	142,7
**MIMAT0000073**	0,0	12,5	4,6	38,0	62,2	9,8	160,6	0,0	154,6
**MIMAT0000074**	0,0	0,0	0,0	28,5	12,0	3,6	0,0	0,0	0,0
**MIMAT0000074_1**	0,0	0,0	4,6	23,8	10,0	6,7	0,0	0,0	0,0
**MIMAT0000075**	622,2	0,0	388,4	262,4	572,9	407,0	998,5	204,3	662,2
**MIMAT0000076**	12204,3	13598,1	26783,7	12613,3	12238,5	11736,6	12871,9	10500,2	11693,2
**MIMAT0000077**	6402,8	162,5	383,7	710,1	744,6	800,0	659,9	171,6	951,6
**MIMAT0000078**	619,2	0,0	0,0	0,0	66,4	1,0	0,0	179,8	0,0
**MIMAT0000080**	532,1	949,9	453,1	958,9	879,0	915,6	269,2	719,1	741,5
**MIMAT0000080_1**	562,1	1212,3	517,8	983,3	937,3	921,7	334,3	408,6	769,2
**MIMAT0000081**	5347,7	3037,1	2991,4	7081,2	8961,0	8260,1	10484,2	7403,2	8441,8
**MIMAT0000082**	10668,3	10985,9	13542,1	11663,3	10360,7	10873,6	8947,4	10737,1	12303,9
**MIMAT0000082_1**	10388,7	11010,9	13093,6	11885,3	10520,4	11136,7	8778,1	11170,2	12684,5
**MIMAT0000083**	1136,3	2149,7	901,6	1188,7	1325,3	1369,5	1697,4	1846,7	2093,6
**MIMAT0000084**	958,9	4699,3	2376,5	1956,4	1907,9	1604,1	1888,5	2304,3	3136,4
**MIMAT0000085**	3,0	12,5	499,3	3,0	41,5	59,8	73,8	8,2	0,0
**MIMAT0000086**	1497,0	1224,8	1021,8	166,8	422,6	305,9	0,0	8,2	460,0
**MIMAT0000087**	766,5	1562,3	3070,0	324,8	382,8	309,5	1224,2	1307,4	154,6
**MIMAT0000088**	171,3	25,0	462,3	54,0	109,8	121,2	112,9	237,0	210,2
**MIMAT0000090**	3,0	0,0	9,2	106,9	91,7	125,9	173,7	326,9	0,0
**MIMAT0000092**	3483,9	3849,5	2774,1	11250,0	9875,9	11006,2	9511,8	8539,0	8628,2
**MIMAT0000092_1**	3387,8	3637,0	2533,7	11067,1	9373,3	10005,5	8860,6	9160,1	8378,4
**MIMAT0000093**	0,0	0,0	0,0	174,6	218,3	314,1	195,4	187,9	230,0
**MIMAT0000094**	0,0	0,0	263,5	44,5	12,0	0,0	0,0	138,9	0,0
**MIMAT0000095**	0,0	0,0	0,0	110,4	91,0	81,0	117,2	106,2	198,3
**MIMAT0000096**	270,5	262,5	18,5	463,1	284,0	430,7	17,4	0,0	170,5
**MIMAT0000097**	5122,2	4311,9	4383,0	3126,2	4063,8	3603,4	2522,3	2672,0	2930,3
**MIMAT0000098**	5876,7	699,9	2353,3	374,7	754,0	743,8	1033,2	359,5	218,1
**MIMAT0000099**	2573,1	674,9	2926,6	2231,4	2840,6	2715,2	2800,1	2598,5	1954,8
**MIMAT0000099_1**	2558,1	787,4	3033,0	2411,9	2949,8	2890,6	3295,0	2672,0	1958,8
**MIMAT0000100**	0,0	0,0	0,0	31,5	5,5	21,1	47,8	0,0	0,0
**MIMAT0000100_1**	0,0	0,0	0,0	26,7	3,9	20,6	78,1	0,0	0,0

**MIMAT0000101**	0,0	412,4	203,4	158,5	130,8	147,5	338,6	163,4	281,5
**MIMAT0000101_1**	3,0	412,4	272,8	155,6	146,7	165,1	343,0	163,4	253,8
**MIMAT0000104**	0,0	12,5	268,2	33,3	37,6	23,7	4,3	8,2	142,7
**MIMAT0000222**	324,6	487,4	425,4	1325,3	1868,0	1844,5	1905,8	2851,8	2589,2
**MIMAT0000227**	0,0	0,0	0,0	0,0	18,1	15,0	0,0	0,0	202,2
**MIMAT0000231**	120,2	12,5	217,3	47,5	54,7	61,4	0,0	0,0	4,0
**MIMAT0000231_1**	135,3	0,0	263,5	51,1	47,6	54,7	4,3	0,0	0,0
**MIMAT0000232**	3,0	287,5	1243,7	37,4	70,3	36,1	104,2	0,0	206,2
**MIMAT0000232_1**	0,0	350,0	1003,3	44,5	71,6	38,7	95,5	16,3	222,0
**MIMAT0000243**	16085,1	25083,9	18124,0	32092,3	47595,1	46380,5	24328,6	27169,7	20908,3
**MIMAT0000244**	201,4	12,5	730,5	239,9	181,4	340,4	191,0	89,9	305,3
**MIMAT0000244_1**	156,3	12,5	776,7	220,3	182,3	346,1	147,6	171,6	230,0
**MIMAT0000245**	6483,9	11348,4	8220,5	10520,9	10798,6	10791,6	6021,4	7632,0	7018,3
**MIMAT0000250**	580,2	774,9	1498,0	216,7	428,5	261,0	790,1	416,7	0,0
**MIMAT0000252**	141,3	562,4	4,6	571,2	518,2	578,7	455,8	433,1	519,4
**MIMAT0000252_1**	177,4	437,4	0,0	554,0	482,6	555,5	421,1	514,8	574,9
**MIMAT0000252_2**	156,3	374,9	4,6	549,2	484,2	551,9	468,9	424,9	491,7
**MIMAT0000253**	6565,1	8348,8	9658,4	4589,2	6370,7	4840,8	6338,3	6929,3	6106,3
**MIMAT0000254**	17732,3	22796,8	20005,7	7267,7	9140,7	8861,0	15099,0	13123,2	15920,1
**MIMAT0000256**	745,5	175,0	850,7	237,5	336,5	292,5	677,2	441,3	559,1
**MIMAT0000256_1**	775,5	275,0	721,3	219,7	326,8	323,9	685,9	441,3	571,0
**MIMAT0000257**	0,0	212,5	0,0	45,7	38,5	81,5	69,5	16,3	4,0
**MIMAT0000257_1**	0,0	200,0	4,6	49,9	39,8	81,0	43,4	89,9	0,0
**MIMAT0000259**	2876,7	474,9	443,9	1875,7	2272,9	1914,1	2926,0	947,9	1526,6
**MIMAT0000261**	2197,4	325,0	1382,4	3275,8	3904,2	3911,8	3898,5	2680,2	2712,2
**MIMAT0000263**	0,0	0,0	0,0	0,0	25,6	0,0	325,6	0,0	0,0
**MIMAT0000264**	523,0	112,5	4,6	15,4	16,5	8,3	0,0	0,0	35,7
**MIMAT0000267**	138,3	0,0	0,0	0,6	5,8	0,0	0,0	8,2	0,0
**MIMAT0000272**	12,0	0,0	9,2	141,3	209,2	145,5	4,3	0,0	7,9
**MIMAT0000275**	3,0	0,0	0,0	42,2	30,1	67,6	0,0	0,0	0,0
**MIMAT0000275_1**	3,0	0,0	0,0	33,8	30,8	62,9	4,3	0,0	0,0
**MIMAT0000278**	3,0	1024,9	4,6	213,8	145,7	107,8	751,0	16,3	0,0
**MIMAT0000279**	643,3	100,0	957,1	1309,8	717,7	984,7	751,0	792,6	571,0
**MIMAT0000280**	0,0	25,0	0,0	35,6	48,6	32,0	0,0	0,0	0,0
**MIMAT0000281**	6,0	0,0	531,7	422,8	454,1	385,8	86,8	0,0	574,9
**MIMAT0000318**	3,0	0,0	0,0	41,6	0,6	0,5	73,8	8,2	0,0
**MIMAT0000414**	7220,4	4224,4	1484,1	6413,2	6583,2	6249,0	7801,3	7893,5	8564,7
**MIMAT0000415**	16947,8	15422,8	17587,6	36271,8	33614,2	34463,4	32624,8	34997,8	38291,5
**MIMAT0000416**	186,4	0,0	4,6	5,9	74,2	16,5	191,0	8,2	0,0
**MIMAT0000416_1**	162,3	0,0	0,0	3,0	79,7	15,5	178,0	8,2	0,0
**MIMAT0000417**	0,0	0,0	0,0	0,0	82,9	6,7	4,3	187,9	0,0
**MIMAT0000418**	0,0	687,4	4,6	28,5	37,6	0,5	4,3	89,9	0,0
**MIMAT0000419**	3315,6	2462,1	1830,9	1830,0	2016,4	1862,0	1888,5	1740,5	2395,0
**MIMAT0000420**	6,0	0,0	0,0	20,8	11,3	0,0	0,0	0,0	0,0
**MIMAT0000421**	96600,6	41831,5	61062,0	82821,0	95959,7	96922,0	15815,3	14945,4	10816,9
**MIMAT0000423**	405,8	0,0	4,6	21,4	79,0	62,9	0,0	0,0	4,0
**MIMAT0000423_1**	375,7	0,0	0,0	19,0	68,0	77,9	0,0	0,0	0,0
**MIMAT0000424**	3655,3	2812,1	1673,7	3534,7	3868,6	3684,4	1654,0	1250,2	2006,4

**MIMAT0000424_1**	2543,1	1074,8	841,5	2156,0	2538,8	2061,7	646,9	735,4	1296,6
**MIMAT0000425**	0,0	0,0	0,0	6,5	26,9	0,0	0,0	0,0	0,0
**MIMAT0000431**	0,0	0,0	0,0	81,3	20,7	58,8	147,6	0,0	333,1
**MIMAT0000433**	42,1	2274,7	2297,9	2323,4	3048,5	2677,0	4063,4	4265,4	4908,9
**MIMAT0000434**	0,0	12,5	0,0	454,8	410,7	426,1	91,2	866,2	456,0
**MIMAT0000435**	6474,9	7948,9	9399,5	1638,2	1791,9	1755,3	6481,5	4298,1	1268,9
**MIMAT0000436**	0,0	399,9	4,6	1490,3	1183,4	1310,7	1115,7	1266,6	1316,4
**MIMAT0000437**	0,0	0,0	402,2	0,0	0,3	0,0	438,5	8,2	321,2
**MIMAT0000438**	0,0	212,5	9,2	50,5	24,6	73,8	0,0	16,3	0,0
**MIMAT0000440**	1536,1	1687,3	887,7	730,3	1088,5	1470,6	1532,5	2353,3	182,4
**MIMAT0000443**	1325,6	3712,0	5071,9	982,1	978,4	728,3	1832,0	1291,1	824,8
**MIMAT0000444**	3,0	674,9	4,6	24,9	186,9	81,0	65,1	8,2	194,3
**MIMAT0000445**	9973,9	31658,0	26349,1	11507,1	13744,5	14782,9	19939,5	25306,6	21059,0
**MIMAT0000446**	24,0	50,0	9,2	55,8	58,9	36,1	138,9	16,3	4,0
**MIMAT0000447**	0,0	12,5	184,9	394,9	276,3	105,2	169,3	0,0	4,0
**MIMAT0000449**	411,8	1312,3	2265,5	1005,8	858,9	1740,8	811,8	1233,9	559,1
**MIMAT0000451**	3,0	387,4	4,6	323,0	519,8	192,9	1002,8	1103,1	1605,9
**MIMAT0000454**	28208,2	25,0	0,0	136,0	20,4	5,2	230,1	490,3	0,0
**MIMAT0000455**	487,0	187,5	23,1	545,7	600,1	501,9	277,8	441,3	812,9
**MIMAT0000456**	682,4	599,9	610,3	1034,9	1139,7	1023,9	555,7	1707,8	1697,1
**MIMAT0000458**	0,0	0,0	0,0	28,5	0,0	19,6	0,0	0,0	0,0
**MIMAT0000460**	0,0	150,0	0,0	89,1	64,1	89,2	160,6	122,6	0,0
**MIMAT0000460_1**	0,0	150,0	9,2	92,0	72,2	86,7	186,7	65,4	0,0
**MIMAT0000461**	0,0	0,0	0,0	0,0	16,8	0,5	0,0	0,0	0,0
**MIMAT0000462**	2278,5	287,5	0,0	0,0	45,0	34,6	0,0	0,0	0,0
**MIMAT0000510**	1196,4	1787,2	1770,8	1732,6	1933,8	2032,3	1059,3	1225,7	1126,1
**MIMAT0000617**	0,0	0,0	0,0	26,1	47,6	28,4	0,0	269,7	0,0
**MIMAT0000646**	348,7	1687,3	1479,5	118,2	88,4	148,6	738,0	0,0	705,8
**MIMAT0000680**	0,0	187,5	0,0	137,8	79,3	66,0	0,0	498,5	0,0
**MIMAT0000681**	3,0	374,9	0,0	42,2	6,8	29,4	0,0	0,0	0,0
**MIMAT0000682**	1424,8	0,0	4,6	17,8	40,8	59,8	4,3	0,0	0,0
**MIMAT0000688**	0,0	0,0	0,0	0,0	17,2	0,0	0,0	0,0	0,0
**MIMAT0000689**	1163,3	1487,3	938,6	425,7	636,1	625,2	616,5	482,1	749,4
**MIMAT0000692**	435,9	512,4	420,7	978,5	759,5	855,2	586,1	604,7	1447,3
**MIMAT0000693**	219,4	512,4	13,9	442,4	486,4	680,9	325,6	106,2	1030,9
**MIMAT0000703**	0,0	0,0	4,6	38,6	18,8	55,2	0,0	0,0	0,0
**MIMAT0000705**	0,0	0,0	0,0	42,8	0,0	28,4	4,3	8,2	0,0
**MIMAT0000707**	276,6	424,9	494,7	1936,3	1935,1	2176,2	1332,8	2410,5	2617,0
**MIMAT0000722**	6,0	25,0	18,5	18,4	20,1	14,4	13,0	16,3	0,0
**MIMAT0000727**	0,0	0,0	0,0	62,3	76,1	105,7	130,2	0,0	222,0
**MIMAT0000728**	279,6	162,5	420,7	188,8	377,3	278,5	4,3	8,2	7,9
**MIMAT0000732**	225,4	62,5	161,8	299,9	287,3	352,3	60,8	187,9	388,6
**MIMAT0000733**	3,0	12,5	4,6	52,3	24,6	24,8	0,0	8,2	4,0
**MIMAT0000736**	0,0	12,5	0,0	43,9	29,5	146,0	8,7	16,3	0,0
**MIMAT0000737**	0,0	0,0	0,0	209,6	236,1	168,2	147,6	0,0	0,0
**MIMAT0000750**	3,0	12,5	0,0	221,5	134,7	160,9	4,3	40,9	0,0
**MIMAT0000751**	0,0	0,0	9,2	96,2	23,6	30,9	0,0	122,6	0,0
**MIMAT0000752**	9,0	0,0	13,9	226,8	184,3	150,1	247,5	8,2	35,7

**MIMAT0000753**	0,0	0,0	1063,4	0,0	0,0	16,0	0,0	849,8	233,9
**MIMAT0000754**	0,0	0,0	0,0	0,0	23,6	0,5	0,0	0,0	0,0
**MIMAT0000755**	0,0	0,0	0,0	0,0	22,0	20,6	0,0	0,0	0,0
**MIMAT0000756**	0,0	0,0	0,0	4,2	32,1	0,0	0,0	0,0	0,0
**MIMAT0000757**	10857,6	8611,3	10726,4	7843,6	9608,1	10209,8	5287,7	4584,1	4361,7
**MIMAT0000759**	745,5	399,9	83,2	399,0	610,8	421,9	17,4	286,0	285,5
**MIMAT0000763**	0,0	0,0	0,0	0,0	0,0	0,0	0,0	0,0	257,7
**MIMAT0000764**	0,0	0,0	0,0	7,7	9,4	36,6	0,0	0,0	0,0
**MIMAT0000772**	0,0	25,0	0,0	11,9	0,6	42,3	0,0	0,0	0,0
**MIMAT0001080**	0,0	0,0	0,0	36,2	0,0	26,8	212,7	179,8	0,0
**MIMAT0001340**	414,8	2337,2	596,4	3095,3	2622,0	2520,2	1775,6	1029,6	1827,9
**MIMAT0001343**	0,0	0,0	0,0	7,7	30,1	0,5	0,0	0,0	222,0
**MIMAT0001413**	0,0	0,0	0,0	7,7	100,7	28,4	0,0	0,0	0,0
**MIMAT0001545**	0,0	0,0	0,0	11,3	12,0	0,0	4,3	0,0	0,0
**MIMAT0001545_1**	0,0	0,0	0,0	8,3	12,0	0,0	0,0	0,0	0,0
**MIMAT0001618**	0,0	0,0	0,0	17,8	0,3	18,1	0,0	0,0	0,0
**MIMAT0001620**	0,0	0,0	13,9	0,0	21,7	0,0	0,0	0,0	0,0
**MIMAT0001621**	0,0	0,0	0,0	7,7	21,4	42,3	0,0	0,0	0,0
**MIMAT0001631**	7436,8	14098,0	6250,9	26772,8	34652,5	35062,7	33809,9	47704,3	38699,9
**MIMAT0001639**	610,2	1287,3	929,3	2115,6	2081,2	1905,9	625,1	514,8	158,6
**MIMAT0002174**	3,0	225,0	13,9	206,0	123,7	174,9	178,0	89,9	4,0
**MIMAT0002175**	3,0	275,0	416,1	163,3	129,5	179,0	0,0	81,7	186,4
**MIMAT0002176**	0,0	362,4	0,0	133,6	114,0	130,5	269,2	0,0	0,0
**MIMAT0002177**	19653,2	9748,6	8151,2	32782,8	34142,1	28120,5	27593,2	23051,3	23743,4
**MIMAT0002177_1**	20134,1	10111,1	8706,0	34112,3	35436,9	29662,8	28570,0	20624,5	24504,7
**MIMAT0002808**	264,5	0,0	0,0	42,8	39,8	25,3	0,0	8,2	0,0
**MIMAT0002809**	2170,3	2249,7	3754,2	2153,0	2227,5	2659,0	6225,4	7713,7	8981,1
**MIMAT0002814**	0,0	0,0	9,2	36,8	0,3	0,5	69,5	0,0	0,0
**MIMAT0002817**	0,0	624,9	0,0	17,2	72,5	71,7	0,0	0,0	0,0
**MIMAT0002821**	0,0	12,5	4,6	0,0	0,3	1,5	0,0	8,2	210,2
**MIMAT0002870**	0,0	0,0	0,0	0,0	27,2	48,0	0,0	0,0	0,0
**MIMAT0002874**	0,0	0,0	0,0	15,4	23,3	35,6	0,0	0,0	4,0
**MIMAT0002875**	0,0	0,0	0,0	16,0	0,0	0,0	208,4	0,0	0,0
**MIMAT0002888**	814,6	1249,8	4,6	899,6	780,8	873,3	898,6	572,0	1225,2
**MIMAT0002891**	0,0	0,0	0,0	35,0	40,2	23,7	0,0	8,2	0,0
**MIMAT0003218**	213,4	25,0	4,6	86,1	103,0	57,3	0,0	179,8	4,0
**MIMAT0003239**	0,0	0,0	0,0	0,0	19,4	0,0	0,0	0,0	0,0
**MIMAT0003241**	0,0	0,0	0,0	0,0	39,2	0,0	0,0	0,0	4,0
**MIMAT0003249**	279,6	62,5	878,5	408,5	392,5	435,9	134,6	40,9	4,0
**MIMAT0003266**	360,7	50,0	0,0	0,0	0,0	18,1	0,0	0,0	0,0
**MIMAT0003297**	0,0	0,0	0,0	71,8	24,6	18,1	0,0	0,0	0,0
**MIMAT0003321**	60,1	0,0	0,0	0,0	24,6	0,0	0,0	0,0	0,0
**MIMAT0003322**	0,0	0,0	0,0	7,7	0,3	0,0	334,3	318,7	0,0
**MIMAT0003329**	0,0	0,0	0,0	21,4	20,7	1,0	0,0	424,9	0,0
**MIMAT0003330**	3,0	212,5	0,0	0,0	9,4	1,5	0,0	0,0	0,0
**MIMAT0003338**	0,0	0,0	0,0	4,8	25,3	25,8	0,0	0,0	0,0
**MIMAT0003339**	3,0	0,0	0,0	14,3	0,3	29,9	0,0	0,0	0,0
**MIMAT0003385**	0,0	0,0	0,0	13,7	0,3	0,0	165,0	0,0	0,0

**MIMAT0003389**	0,0	0,0	0,0	0,0	22,7	0,0	0,0	0,0	0,0
**MIMAT0003393**	6,0	37,5	9,2	238,1	196,3	185,7	182,3	16,3	134,8
**MIMAT0003884**	0,0	0,0	0,0	19,0	0,0	15,0	0,0	0,0	0,0
**MIMAT0003886**	3,0	12,5	0,0	41,6	23,6	1,5	0,0	0,0	0,0
**MIMAT0004481**	0,0	0,0	46,2	32,1	103,6	83,6	65,1	0,0	4,0
**MIMAT0004481_1**	0,0	0,0	64,7	40,4	104,3	53,1	91,2	0,0	4,0
**MIMAT0004482**	0,0	0,0	0,0	36,2	48,3	29,9	91,2	0,0	0,0
**MIMAT0004484**	3649,3	1287,3	980,2	2600,7	3405,1	2048,8	1319,8	1062,3	1859,7
**MIMAT0004495**	72,1	0,0	0,0	32,7	10,4	23,7	0,0	0,0	0,0
**MIMAT0004497**	0,0	0,0	0,0	39,2	52,5	22,2	0,0	8,2	182,4
**MIMAT0004498**	3,0	0,0	0,0	0,0	0,0	9,8	0,0	8,2	130,9
**MIMAT0004500**	0,0	0,0	0,0	15,4	0,3	0,0	134,6	0,0	0,0
**MIMAT0004501**	0,0	0,0	0,0	22,0	0,0	13,9	0,0	114,4	122,9
**MIMAT0004502**	3,0	0,0	9,2	48,7	212,1	252,2	460,2	482,1	289,5
**MIMAT0004505**	0,0	0,0	0,0	0,0	24,6	0,0	0,0	0,0	0,0
**MIMAT0004518**	2991,0	1137,3	1655,2	2582,9	2438,0	2423,8	2470,2	2255,3	2843,0
**MIMAT0004549**	0,0	0,0	0,0	0,0	17,5	0,0	0,0	0,0	0,0
**MIMAT0004552**	330,7	0,0	0,0	113,4	64,8	99,5	4,3	147,1	0,0
**MIMAT0004558**	0,0	12,5	0,0	33,8	47,0	78,9	0,0	0,0	0,0
**MIMAT0004563**	3,0	399,9	1188,2	35,0	68,7	34,0	104,2	8,2	206,2
**MIMAT0004568**	0,0	0,0	0,0	0,0	8,7	51,1	0,0	0,0	0,0
**MIMAT0004570**	327,7	537,4	887,7	368,1	694,7	537,5	507,9	57,2	384,6
**MIMAT0004571**	0,0	0,0	0,0	0,0	17,8	0,0	0,0	0,0	0,0
**MIMAT0004586**	0,0	0,0	0,0	22,6	0,3	76,9	0,0	0,0	0,0
**MIMAT0004594**	0,0	0,0	4,6	0,0	26,6	21,7	0,0	0,0	0,0
**MIMAT0004597**	1611,2	899,9	1160,5	548,6	987,1	778,9	764,1	1470,8	900,1
**MIMAT0004600**	838,7	637,4	0,0	364,0	837,2	624,6	4,3	433,1	400,5
**MIMAT0004601**	0,0	0,0	0,0	64,7	50,2	30,4	0,0	237,0	0,0
**MIMAT0004608**	0,0	0,0	0,0	0,0	23,6	0,0	0,0	0,0	0,0
**MIMAT0004610**	3,0	150,0	0,0	144,9	83,9	115,0	112,9	196,1	499,6
**MIMAT0004614**	0,0	0,0	4,6	83,7	79,0	62,4	4,3	0,0	0,0
**MIMAT0004615**	769,5	0,0	0,0	20,2	0,0	21,1	0,0	0,0	23,8
**MIMAT0004672**	378,8	12,5	13,9	315,9	242,3	336,3	308,2	196,1	7,9
**MIMAT0004678**	0,0	0,0	4,6	23,2	8,1	24,8	0,0	0,0	0,0
**MIMAT0004680**	261,5	0,0	198,8	171,0	227,0	172,3	0,0	73,5	0,0
**MIMAT0004682**	3,0	25,0	13,9	18,4	102,3	213,5	494,9	171,6	0,0
**MIMAT0004688**	0,0	0,0	0,0	57,0	76,1	101,6	0,0	237,0	0,0
**MIMAT0004692**	9,0	300,0	148,0	552,2	336,5	408,0	377,7	24,5	709,8
**MIMAT0004693**	0,0	0,0	0,0	35,6	31,1	15,0	0,0	0,0	0,0
**MIMAT0004694**	1355,7	12,5	1308,4	403,2	381,5	330,6	885,6	1307,4	678,0
**MIMAT0004699**	0,0	0,0	0,0	10,1	58,6	57,8	0,0	0,0	0,0
**MIMAT0004700**	0,0	0,0	0,0	0,0	19,4	13,9	0,0	0,0	0,0
**MIMAT0004701**	0,0	0,0	0,0	0,0	57,6	0,0	0,0	0,0	0,0
**MIMAT0004702**	0,0	0,0	0,0	30,9	22,0	80,5	0,0	73,5	0,0
**MIMAT0004703**	6,0	0,0	13,9	136,0	73,2	48,5	8,7	0,0	0,0
**MIMAT0004748**	11302,5	6111,6	4498,6	15829,1	16968,2	17199,5	10527,6	9797,4	11340,3
**MIMAT0004749**	3,0	125,0	4,6	65,3	88,1	69,1	321,3	0,0	206,2
**MIMAT0004761**	0,0	12,5	0,0	306,4	456,0	339,9	0,0	0,0	218,1

**MIMAT0004762**	0,0	12,5	0,0	126,5	226,7	117,6	178,0	114,4	71,4
**MIMAT0004762_1**	3,0	0,0	4,6	101,5	93,9	61,4	117,2	171,6	63,4
**MIMAT0004767**	117,2	0,0	0,0	42,8	63,2	44,9	0,0	0,0	0,0
**MIMAT0004774**	3,0	12,5	4,6	57,6	105,6	164,5	0,0	171,6	0,0
**MIMAT0004775**	0,0	0,0	0,0	0,0	0,0	30,4	21,7	0,0	0,0
**MIMAT0004776**	0,0	0,0	0,0	11,9	40,5	16,5	0,0	0,0	0,0
**MIMAT0004792**	0,0	0,0	0,0	86,1	24,6	51,6	0,0	130,7	0,0
**MIMAT0004796**	0,0	0,0	0,0	4,8	8,4	8,8	0,0	8,2	0,0
**MIMAT0004797**	0,0	0,0	0,0	0,0	22,3	0,0	0,0	155,3	0,0
**MIMAT0004799**	0,0	0,0	0,0	19,0	0,3	25,8	4,3	0,0	4,0
**MIMAT0004808**	3,0	0,0	235,8	216,7	197,6	184,7	8,7	0,0	170,5
**MIMAT0004810**	3,0	0,0	4,6	105,7	211,2	69,6	151,9	8,2	0,0
**MIMAT0004813**	0,0	0,0	0,0	0,0	17,8	21,7	0,0	0,0	0,0
**MIMAT0004814**	3,0	0,0	554,8	37,4	52,8	44,4	0,0	0,0	4,0
**MIMAT0004819**	387,8	25,0	4,6	68,3	109,8	77,9	0,0	8,2	0,0
**MIMAT0004920**	0,0	0,0	0,0	0,0	16,2	16,0	0,0	0,0	0,0
**MIMAT0004921**	0,0	12,5	0,0	11,3	29,5	19,6	0,0	0,0	0,0
**MIMAT0004928**	0,0	0,0	0,0	0,0	20,7	0,0	0,0	0,0	0,0
**MIMAT0004945**	21,0	50,0	411,5	408,5	406,8	362,6	4,3	269,7	7,9
**MIMAT0004947**	0,0	0,0	0,0	0,0	13,0	15,5	0,0	0,0	0,0
**MIMAT0004949**	0,0	0,0	50,9	13,7	13,6	11,9	0,0	0,0	31,7
**MIMAT0004954**	336,7	474,9	32,4	979,7	850,1	859,8	243,1	24,5	0,0
**MIMAT0004957**	3,0	0,0	4,6	72,4	1,9	69,1	0,0	0,0	0,0
**MIMAT0004959**	0,0	0,0	0,0	0,0	20,1	0,0	0,0	0,0	0,0
**MIMAT0004976**	0,0	0,0	0,0	0,0	29,5	0,0	0,0	0,0	0,0
**MIMAT0004982**	0,0	0,0	351,4	0,0	13,0	16,0	0,0	0,0	0,0
**MIMAT0004984**	3,0	12,5	0,0	22,0	29,8	30,9	0,0	8,2	35,7
**MIMAT0004984_1**	3,0	25,0	0,0	23,8	31,1	27,3	0,0	0,0	39,7
**MIMAT0004984_2**	0,0	50,0	0,0	19,6	26,6	32,5	0,0	8,2	39,7
**MIMAT0004984_3**	0,0	50,0	0,0	18,4	30,1	30,9	0,0	0,0	27,8
**MIMAT0004984_4**	3,0	62,5	0,0	17,8	32,4	35,6	4,3	0,0	39,7
**MIMAT0004985**	0,0	0,0	0,0	66,5	59,3	28,4	4,3	0,0	0,0
**MIMAT0005458**	0,0	0,0	0,0	13,7	0,0	13,9	0,0	0,0	0,0
**MIMAT0005580**	0,0	0,0	0,0	0,0	21,4	0,0	0,0	0,0	0,0
**MIMAT0005582**	174,3	0,0	448,5	35,6	20,4	34,0	0,0	0,0	0,0
**MIMAT0005792**	0,0	12,5	37,0	80,2	110,1	150,6	17,4	228,8	83,3
**MIMAT0005792_1**	0,0	0,0	27,7	85,5	99,8	163,0	13,0	237,0	75,3
**MIMAT0005793**	0,0	0,0	0,0	46,9	55,4	73,2	4,3	24,5	35,7
**MIMAT0005793_1**	0,0	0,0	4,6	45,1	54,4	78,9	4,3	8,2	27,8
**MIMAT0005797**	0,0	0,0	9,2	73,0	88,7	1,5	0,0	204,3	0,0
**MIMAT0005825**	0,0	25,0	13,9	149,6	139,9	80,5	199,7	0,0	154,6
**MIMAT0005874**	0,0	0,0	0,0	0,0	24,6	0,0	0,0	0,0	0,0
**MIMAT0005884**	0,0	0,0	4,6	23,8	21,7	14,4	0,0	0,0	0,0
**MIMAT0005919**	0,0	0,0	0,0	0,0	13,9	11,3	0,0	8,2	0,0
**MIMAT0005919_1**	0,0	0,0	0,0	0,0	15,2	7,2	0,0	0,0	0,0
**MIMAT0005933**	0,0	0,0	0,0	0,0	27,9	0,0	0,0	0,0	0,0
**MIMAT0005936**	0,0	0,0	0,0	28,5	35,3	15,5	52,1	0,0	71,4
**MIMAT0005943**	0,0	0,0	0,0	0,0	12,3	18,6	0,0	130,7	4,0

**MIMAT0005948**	3,0	0,0	18,5	0,0	25,3	16,5	0,0	0,0	0,0
**MIMAT0005950**	0,0	37,5	0,0	16,6	21,1	0,0	0,0	0,0	0,0
**MIMAT0005951**	330,7	762,4	32,4	530,8	302,8	417,3	199,7	220,6	222,0
**MIMAT0006764**	0,0	0,0	0,0	25,5	11,3	15,0	8,7	24,5	7,9
**MIMAT0006764_1**	0,0	0,0	0,0	25,5	11,0	13,4	0,0	8,2	11,9
**MIMAT0006789**	0,0	0,0	4,6	0,0	21,4	17,5	65,1	204,3	0,0
**MIMAT0007881**	0,0	0,0	0,0	5,3	15,5	0,0	0,0	0,0	0,0
**MIMAT0010133**	468,9	0,0	0,0	24,9	49,2	37,1	134,6	8,2	4,0
**MIMAT0015006**	0,0	0,0	0,0	0,0	0,0	45,4	0,0	0,0	0,0
**MIMAT0015032**	315,6	0,0	4,6	87,9	78,4	134,6	4,3	49,0	249,8
**MIMAT0015032_1**	321,6	0,0	0,0	78,4	80,0	133,6	4,3	65,4	297,4
**MIMAT0015043**	51,1	75,0	203,4	12,5	9,7	22,7	91,2	130,7	138,8
**MIMAT0015050**	0,0	0,0	4,6	87,3	268,8	69,6	0,0	0,0	230,0
**MIMAT0016888**	0,0	12,5	0,0	0,0	23,0	19,6	0,0	0,0	0,0
**MIMAT0017981**	0,0	0,0	0,0	0,0	13,9	36,6	0,0	0,0	0,0
**MIMAT0017982**	0,0	0,0	9,2	42,8	81,9	21,7	0,0	0,0	0,0
**MIMAT0017990**	0,0	0,0	0,0	58,2	17,8	36,6	99,8	8,2	0,0
**MIMAT0017994**	174,3	399,9	32,4	410,3	256,8	415,7	182,3	539,3	186,4
**MIMAT0018104**	0,0	0,0	0,0	5,3	16,5	32,5	0,0	0,0	0,0
**MIMAT0018187_1**	0,0	0,0	0,0	0,0	11,3	0,0	43,4	65,4	0,0
**MIMAT0018965**	0,0	325,0	0,0	12,5	21,1	97,0	0,0	16,3	4,0
**MIMAT0019000**	0,0	0,0	0,0	0,0	17,8	0,0	143,3	0,0	0,0
**MIMAT0019210**	0,0	0,0	0,0	0,0	21,7	0,0	0,0	0,0	0,0
**MIMAT0019214**	0,0	0,0	0,0	0,0	17,5	0,0	0,0	0,0	0,0
**MIMAT0019226**	2002,0	0,0	0,0	0,0	0,0	0,5	0,0	0,0	0,0
**MIMAT0019743**	0,0	0,0	0,0	0,0	16,8	0,0	0,0	0,0	0,0
**MIMAT0019772**	0,0	0,0	0,0	0,0	17,5	0,0	0,0	0,0	0,0
**MIMAT0019855**	3,0	0,0	0,0	3,6	0,3	34,0	0,0	0,0	198,3
**MIMAT0019856**	0,0	0,0	166,4	0,0	37,9	11,9	34,7	24,5	0,0
**MIMAT0019880**	0,0	0,0	0,0	0,0	21,4	17,0	0,0	0,0	0,0
**MIMAT0020924**	0,0	0,0	0,0	0,0	24,0	6,2	0,0	0,0	0,0
**MIMAT0021043**	0,0	0,0	0,0	0,0	18,1	16,5	0,0	0,0	0,0
**MIMAT0022693**	0,0	0,0	0,0	12,5	16,2	17,5	0,0	416,7	0,0
**MIMAT0022710**	0,0	0,0	0,0	0,0	19,4	0,0	169,3	0,0	0,0
**MIMAT0022714**	3,0	0,0	4,6	10,7	16,8	0,5	0,0	0,0	0,0
**MIMAT0022727**	0,0	0,0	0,0	19,6	12,6	0,0	0,0	0,0	174,5
**MIMAT0026482**	0,0	0,0	0,0	0,0	23,3	0,0	0,0	0,0	0,0
**MIMAT0027369**	0,0	0,0	0,0	0,0	46,0	15,0	0,0	0,0	154,6
**MIMAT0027384**	0,0	0,0	0,0	52,3	0,0	23,7	0,0	0,0	210,2
**MIMAT0027395**	0,0	0,0	0,0	16,6	42,4	0,0	0,0	0,0	0,0
**MIMAT0027445**	0,0	0,0	0,0	20,8	0,0	12,4	0,0	0,0	0,0
**MIMAT0027513**	0,0	0,0	0,0	0,0	20,1	0,0	0,0	0,0	0,0
**MIMAT0027530**	0,0	0,0	0,0	20,2	10,4	0,0	0,0	0,0	0,0
**MIMAT0027539**	0,0	0,0	0,0	0,0	21,1	0,0	0,0	0,0	0,0
**MIMAT0027587**	0,0	437,4	4,6	27,9	104,3	82,5	0,0	0,0	0,0
**MIMAT0027616**	0,0	0,0	0,0	0,0	18,5	0,0	0,0	0,0	0,0
**MIMAT0027637**	291,6	0,0	0,0	0,0	25,9	0,0	0,0	0,0	0,0
**MIMAT0027689**	0,0	0,0	0,0	20,8	0,0	9,3	99,8	0,0	0,0

**MIMAT0030021**	0,0	0,0	0,0	21,4	19,8	5,2	156,3	0,0	0,0
**MIMAT0030413**	0,0	12,5	0,0	54,6	56,7	64,0	0,0	0,0	0,0
**MIMAT0030414**	1509,0	1424,8	1262,2	989,2	1203,5	1256,0	473,2	49,0	83,3
**MIMAT0039764**	0,0	0,0	0,0	102,1	9,7	99,5	4,3	212,5	0,0
**MIMAT0044657**	0,0	0,0	0,0	7,1	21,1	3,1	0,0	0,0	0,0

[a] Accession number of miRNAs in miRbase (the microRNA database) (https://www.mirbase.org/index.shtml)

[b] Size exclusion chromatography.

[c] Isopycnic ultracentrifugation using iodixanol gradients.

[d] Size exclusion chromatography and isopycnic ultracentrifugation using iodixanol gradients.
